# Lysosomal alterations and decreased electrophysiological activity in CLN3 disease patient-derived cortical neurons

**DOI:** 10.1242/dmm.049651

**Published:** 2022-12-13

**Authors:** Sueanne Chear, Sharn Perry, Richard Wilson, Aidan Bindoff, Jana Talbot, Tyson L. Ware, Alexandra Grubman, James C. Vickers, Alice Pébay, Jonathan B. Ruddle, Anna E. King, Alex W. Hewitt, Anthony L. Cook

**Affiliations:** ^1^ Wicking Dementia Research and Education Centre, University of Tasmania, Hobart, TAS 7001, Australia; ^2^ Central Science Laboratory, University of Tasmania, Hobart, TAS 7001, Australia; ^3^ Department of Paediatrics, Royal Hobart Hospital, Hobart, TAS 7000, Australia; ^4^ Department of Anatomy and Developmental Biology, Monash University, Clayton, VIC 3800, Australia; ^5^ Department of Anatomy and Physiology, University of Melbourne, Parkville, VIC 3010, Australia; ^6^ Department of Surgery, Royal Melbourne Hospital, University of Melbourne, Parkville, VIC 3010, Australia; ^7^ Centre for Eye Research Australia, Royal Victorian Eye and Ear Hospital, East Melbourne, VIC 3002, Australia; ^8^ Menzies Institute for Medical Research, University of Tasmania, Hobart, TAS 7001, Australia

**Keywords:** Juvenile neuronal ceroid lipofuscinosis, Batten disease, Dementia, Induced pluripotent stem cells, Microelectrode array, Proteomics

## Abstract

CLN3 disease is a lysosomal storage disorder associated with fatal neurodegeneration that is caused by mutations in *CLN3*, with most affected individuals carrying at least one allele with a 966 bp deletion. Using CRISPR/Cas9, we corrected the 966 bp deletion mutation in human induced pluripotent stem cells (iPSCs) of a compound heterozygous patient (*CLN3* Δ 966 bp and E295K). We differentiated these isogenic iPSCs, and iPSCs from an unrelated healthy control donor, to neurons and identified disease-related changes relating to protein synthesis, trafficking and degradation, and in neuronal activity, which were not apparent in CLN3-corrected or healthy control neurons. CLN3 neurons showed numerous membrane-bound vacuoles containing diverse storage material and hyperglycosylation of the lysosomal LAMP1 protein. Proteomic analysis showed increase in lysosomal-related proteins and many ribosomal subunit proteins in CLN3 neurons, accompanied by downregulation of proteins related to axon guidance and endocytosis. CLN3 neurons also had lower electrophysical activity as recorded using microelectrode arrays. These data implicate inter-related pathways in protein homeostasis and neurite arborization as contributing to CLN3 disease, and which could be potential targets for therapy.

## Teaser

**Summary:** We used proteomics and electrophysiology to investigate how the common 1 kb deletion in CLN3 affects induced pluripotent stem cell-derived neuronal functions, and suggest pathways that may point to new therapeutic opportunities.

## INTRODUCTION

CLN3 disease (also known as Batten disease or juvenile neuronal ceroid lipofuscinosis) is a lysosomal storage disorder caused by mutations in *CLN3*. There are more than 90 *CLN3* gene mutations reported in the neuronal ceroid lipofuscinosis database (https://www.ucl.ac.uk/ncl-disease/), causing frameshift with a premature stop codon, nonsense mutations, splice defects and missense mutations (Gardner and Mole, 2021). The most prevalent mutation is a 966 bp deletion spanning exons 7 and 8, often referred to as a 1 kb deletion (Lerner et al., 1995; Golabek et al., 1999). Approximately 75% of patients are homozygous for this mutation, and ∼20% of patients are compound heterozygous for this and one of the other rare mutations (Langin et al., 2020).

*CLN3* encodes a 438-amino acid protein, which is predicted to be a hydrophobic six-transmembrane domain protein (Haskell et al., 2000). The role of CLN3 in healthy and disease cells remains poorly understood, despite the identification of *CLN3* as the causative gene for CLN3 disease more than 20 years ago (Lerner et al., 1995). Studies from lower-mammalian and other eukaryotic models have suggested that CLN3 is linked to endocytic trafficking (Schultz et al., 2014), autophagy (Cao et al., 2006), cytoskeletal organization (Parviainen et al., 2017), osmoregulation (Mathavarajah et al., 2018), cell migration (Getty et al., 2011), cell viability (Mathavarajah et al., 2018) and lipid processing (Cotman and Staropoli, 2012; Laqtom et al., 2022). The hallmark of CLN3 disease is the formation of autofluorescent lipopigments with fingerprint profiles, which are enriched in subunit C of mitochondrial ATP synthase (Palmer et al., 1995; Cotman et al., 2002); however, the underlying molecular basis behind subunit C accumulation remains unknown.

CLN3 disease typically presents early in children between 4 and 8 years of age, and is marked by vision failure, cognitive decline, behavioral changes, motor deficit, refractory seizures and eventually death (Ouseph et al., 2016; Pérez-Poyato et al., 2011). The neurological features are accompanied by reductions in whole-brain and cerebral cortex volume mainly due to cortical and cerebellar atrophy and increased cerebrospinal fluid volume (Boustany, 1992; Autti et al., 1996). Loss of Purkinje cells and granular cells in the cerebellum and dentate nucleus (Anderson et al., 2013), neuron loss and glial activation in the hippocampus have also been described in CLN3 post-mortem brain tissue (Tyynelä et al., 2004). Characterization of cellular and molecular alterations can be provided by post-mortem tissue; however, these are usually limited to the late-stage pathological changes, are not amenable to intervention in the course of disease and do not provide longitudinal data. Mouse models are valuable in uncovering disease mechanisms and providing insights into the function of specific genes, especially in neurodegenerative disorders; however, the findings have rarely translated to human therapeutics (Dragunow, 2008).

For these reasons, human induced pluripotent stem cells (iPSCs) have emerged as a powerful tool for disease modeling and drug screening. When combined with CRISPR/Cas9 gene editing, patient-specific isogenic iPSCs can be generated and differentiated into neurons to model CLN3 disease, thus overcoming genetic variability between different donors as confounding factors (Kyttälä et al., 2016; Kilpinen et al., 2017). More importantly, these patient-specific iPSC-derived models can provide insights into the precise roles of mutation and genetic background in the progression of disease. This is especially relevant in CLN3 disease, as even among CLN3 disease-affected family members with identical mutations, variations in symptomatology are observed, possibly owing to genetic and environmental factors (Cooper et al., 2006). The differentiation of patient-specific iPSCs into neurons provides access to human neurons, one of the cell types relevant to neurological manifestations in CLN3 patients. Studies have demonstrated the relevance of iPSC-differentiated neurons harboring CLN3 homozygous and compound heterozygous 966 bp deletion in recapitulating certain CLN3 phenotypes such as fingerprint deposits and accumulation of subunit C of mitochondrial ATP synthase (Kinarivala et al., 2020; Lojewski et al., 2014). In this study, isogenic CLN3 iPSCs derived from a compound heterozygous patient (*CLN3* Δ 966 bp and E295K) were differentiated into neurons to generate a robust disease model to investigate early-stage neuropathology. Our findings provide further insight into lysosomal and neurodevelopmental alterations caused by a CLN3 mutation.

## RESULTS

### Generation of patient-specific CLN3 iPSCs

We reprogrammed fibroblasts from a CLN3 patient to pluripotency under feeder-free conditions using episomal vectors. A series of quality control tests was performed on the reprogrammed iPSC line. CLN3 iPSCs were integration free by passage 7, as demonstrated by PCR on DNA using EBNA1-specific primers (Fig. S1A). Immunocytochemistry (Fig. S1B) results showed that, at the protein level, CLN3 iPSCs expressed pluripotent markers. The Taqman Scorecard analysis demonstrated that the expression levels of pluripotency genes in iPSCs were comparable to those in undifferentiated embryonic stem cells, whereas the iPSC-differentiated embryoid bodies (EBs) displayed an expression pattern of trilineage genes comparable to that of germline-specific controls (Fig. S1C). Karyotyping of reprogrammed iPSCs revealed a normal male karyotype (46, XY) (Fig. S2). Short tandem repeat (STR) analysis of ten different genomic loci indicated that the CLN3 iPSC line was 100% matched to the donor fibroblast DNA.

### CRISPR/Cas9-mediated correction of 966 bp deletion in *CLN3* gene

**Fig. 1.**
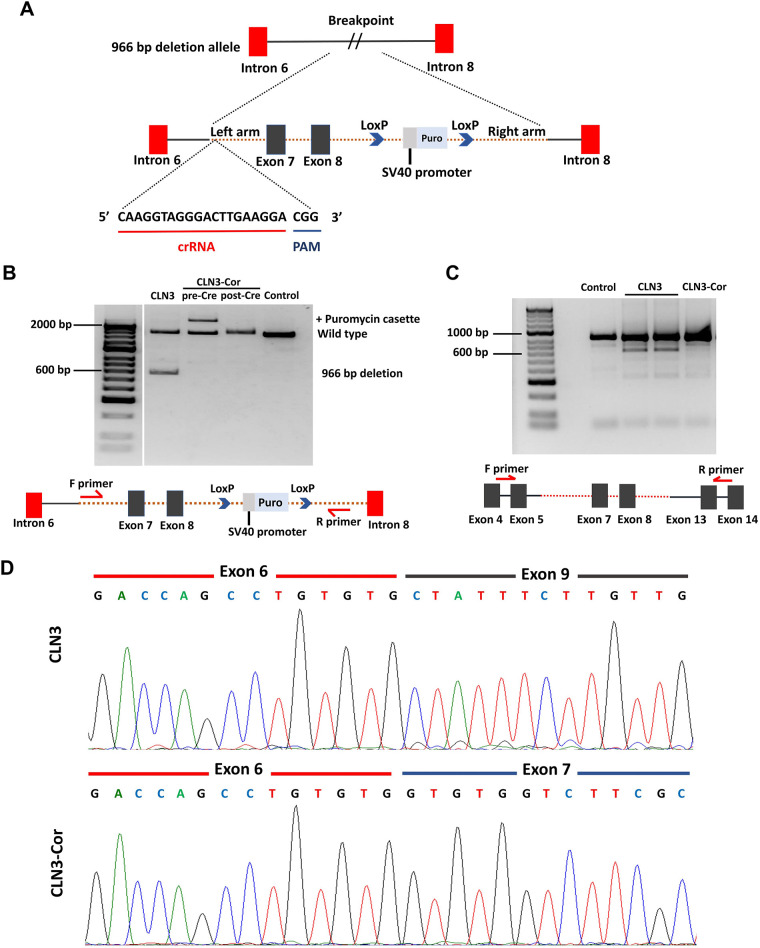
**CRISPR/Cas9-mediated insertion of 966 bp sequence.** (A) Schematic representation depicting donor repair construct carrying the reference CLN3 sequence and a LoxP-flanked puromycin resistance cassette targeted to breakpoint sequence by sgRNA. (B) Genomic PCR products of CLN3 induced pluripotent stem cells (iPSCs), CLN3-corrected (CLN3-Cor) iPSCs before and after Cre-Lox recombination, and a positive control, amplified using primers that span exons 7 and 8 of the *CLN3* gene. (C) RT-PCR of positive control, CLN3 iPSCs and CLN3-Cor iPSCs. Diagrams below gel images depict locations of primers. (D) Sanger sequencing of target cDNA regions revealed restoration of the deleted exons.

To correct the 966 bp deletion mutation, we nucleofected CLN3 iPSCs with an allele-specific single-guide RNA (sgRNA), which targets the breakpoint sequence of the 966 bp deletion allele together with a homology-directed repair (HDR) donor plasmid carrying the reference 966 bp sequence and a floxed puromycin resistance gene for selection (Fig. 1A). Puromycin-resistant clones were validated for HDR correction via PCR using primer sets that target the region between intron 6 and intron 8 of *CLN3*. From PCR gel analysis, 966 bp correction was evident by the larger size of the amplicon, ∼2.5 kbp, which includes the puromycin sequence and the absence of the truncated 966 bp deletion band (Fig. 1B). To avoid alteration in the endogenous DNA sequence after HDR correction of CLN3 iPSCs, the corrected clone was treated with Cre recombinase to excise the puromycin cassette. Following Cre-Lox recombination, the corrected clone demonstrated a single CLN3 band similar to that of a control cell line, along with absence of the ∼2.5 kbp band on PCR (Fig. 1B). PCR of cDNA from the corrected clone also showed a homozygous CLN3 band similar to that of a control cell line (Fig. 1C). Sanger sequencing of the corrected clone cDNA showed restoration of exons 7 and 8 (Fig. 1D). The corrected cell line is hereafter referred to as CLN3-Cor. The CLN3-Cor iPSCs stained positive for pluripotency markers OCT4, SOX2, NANOG, TRA-1-60, TRA-1-81 and SSEA-4 (Fig. S3A). CLN3-Cor iPSC-derived EBs were able to express markers of all three germ layers – mesoderm, ectoderm and endoderm – as demonstrated by Taqman hPSC Scorecard assay (Fig. S3B). Virtual karyotyping analysis from both QuantiSNP and PennCNV methods showed normal male karyotype for CLN3-Cor iPSCs (46, XY) without CRISPR/Cas9 editing-induced chromosomal abnormalities (Fig. S4), and STR analysis confirmed identity to the parental iPSC cell line. Results from Synthego Ice Analysis Tool (Table S1) did not reveal any sgRNA-induced modifications on the predicted off-target sites.

### CLN3 mutation has a minor effect on differentiation efficiency of CLN3-deficient neurons

**Fig. 2.**
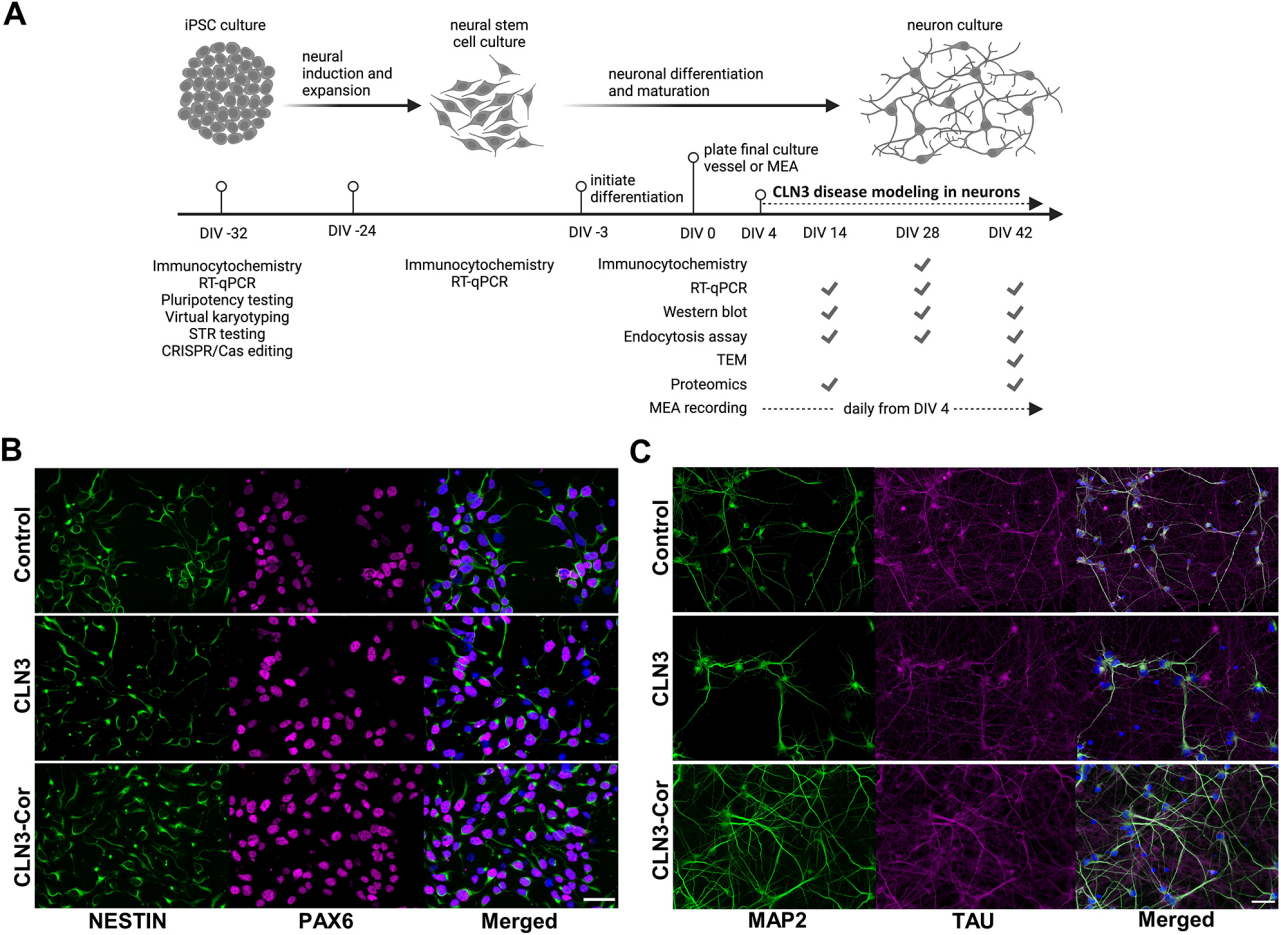
**Neural differentiation of control and isogenic CLN3 iPSCs.** (A) Schematic of differentiation timeline, indicating the timing of key steps and analyses undertaken in this study. This panel was created using biorender.com. (B) Control, CLN3 and CLN3-Cor neural stem cells (NSCs) were positive for NSC markers, NES and PAX6. (C) Control, CLN3 and CLN3-Cor neurons at day *in vitro* (DIV) 28 expressed mature neuronal markers, MAP2 and TAU. Scale bars: 50 µm. For B and C, merged panel also shows nuclei stained with DAPI (blue). MEA, multielectrode array; RT-qPCR, reverse transcription quantitative real-time PCR; STR, short tandem repeat; TEM, transmission electron microscopy.

We next tested whether CLN3 and CLN3-Cor iPSC would differentiate into cortical neurons, and whether features of CLN3 disease were captured in this model (Fig. 2A). CLN3-Cor iPSCs and CLN3 iPSCs, which were derived from single-cell clones, were induced into neural stem cells (NSCs) that expressed PAX6 and NES (Fig. 2B). We then generated three independent cultures of human cortical neuron cultures differentiated from CLN3 isogenic NSCs. The neuronal culture stained positive for mature neuronal markers, MAP2 and TAU (MAPT) (Fig. 2C). Reverse transcription quantitative real-time PCR (RT-qPCR) on RNA extracts from NSCs for both cell lines showed high expression of *NES* and *PAX6*, which then was reduced following neural differentiation (Fig. S5). Both isogenic neuronal cultures showed expression of immature (*TUBB3*) and mature (*MAP2*) neuronal markers, glutamatergic neuron-related genes (*GRIA2*, *GRIN1*, *SLC1A7*, *SLC1A2* and *SLC1A3*), GABAergic neuron-related genes (*GAD1*, *GAD2*) and post-synaptic marker (*DLG4*) (Fig. S5). At day *in vitro* (DIV) 7, the expression of *GAD1*, *GAD2*, *GRIA2*, *GRIN1*, *SLC1A2* and *DLG4* was higher in CLN3 neurons than in CLN3-Cor neurons, whereas the expression of *SLC1A3* was lower in CLN3 neurons than in CLN3-Cor neurons (Fig. S5). At DIV 28, the expression of *MAP2*, *GAD2*, *GRIA2*, *SLC1A2* and *DLG4* was lower in CLN3 neurons than in CLN3-Cor neurons (Fig. S5). The CLN3-Cor cell line had higher expression of *CLN3* at several stages, including iPSCs, NSCs and neurons at DIV 28 (Fig. S5).

A genetically unrelated healthy control cell line was differentiated into neurons alongside the isogenic CLN3 cell lines (again using three independent cultures). The overall patterns of mRNA expression of many neuronal genes in this control and both isogenic CLN3 cell lines were largely similar and consistent with previous studies (Fig. S5) (Togo et al., 2021; Muñoz et al., 2020). Specifically, the expression profiles of neuronal genes throughout the differentiation period in the isogenic CLN3 lines were similar despite minor differences in gene expression at DIV 7 and DIV 28. Reflecting the different genetic backgrounds of the control and CLN3 fibroblast donors, the mRNA expression profiles of the isogenic CLN3 lines were more similar than those of the control and isogenic CLN3 lines (Fig. S6).

### LAMP1 hyperglycosylation, storage accumulation and decreased endocytosis in CLN3 neurons

**Fig. 3.**
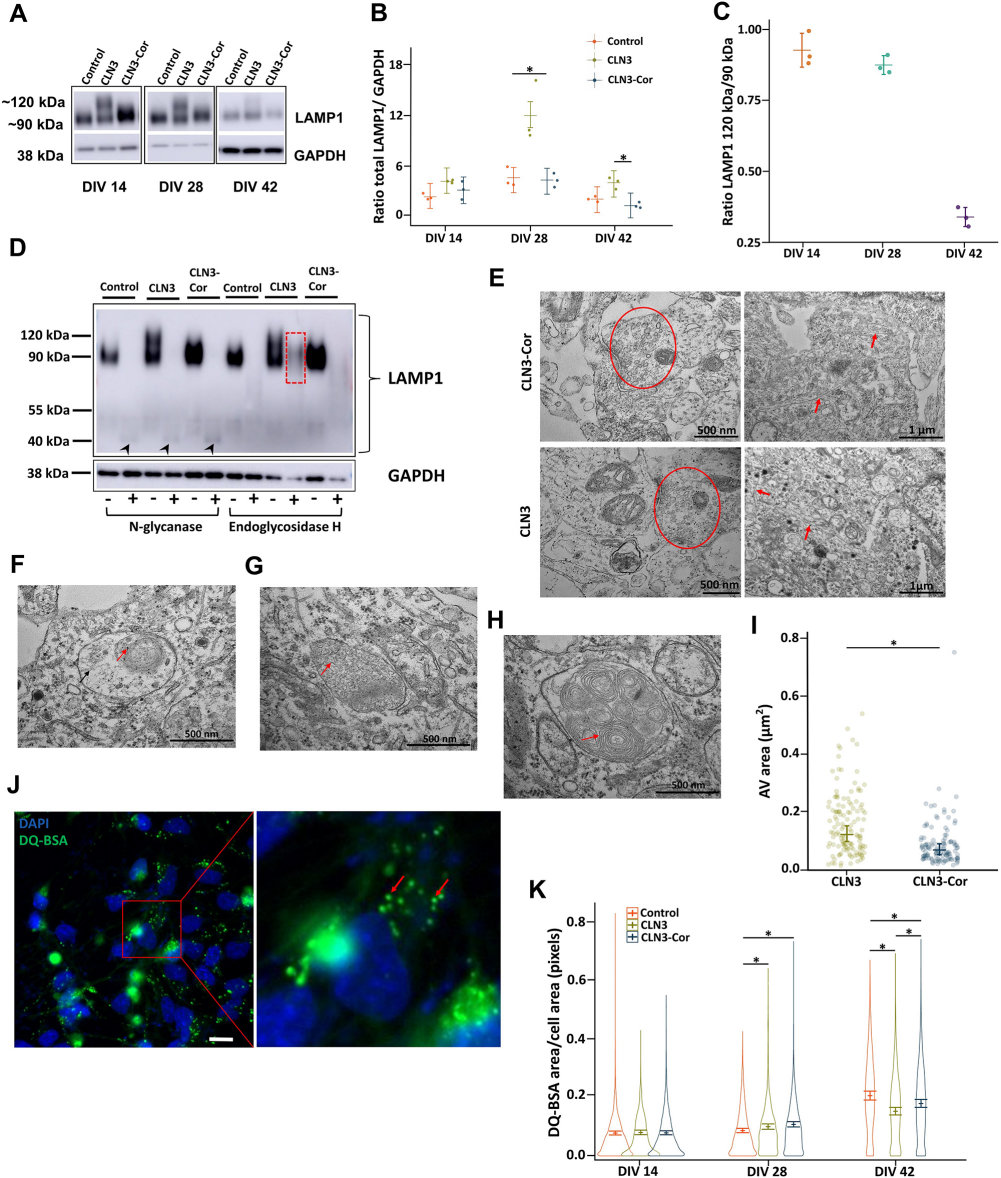
**Alterations in LAMP1 expression, storage material and endocytosis.** (A) Representative western blots showing LAMP1 protein expression across different time points, DIV 14, 28 and 42. Bands corresponding to the higher-molecular mass (∼120 kDa) form of LAMP1 protein were present only in the CLN3 neurons. (B) Expression of total LAMP1 in all cell lines (*n*=3 independent differentiated cultures per cell line per time point; linear mixed-effect model). (C) LAMP1 upper band/lower band expression level ratio in CLN3 neurons across different time points. Data are represented as mean±s.d. (D) LAMP1 protein expression after treatment with or without N-glycanase and endoglycosidase H digestion. The N-glycanase-deglycosylated form of LAMP1 is highlighted by arrowheads. Diffuse LAMP1 band in CLN3 neurons after endoglycosidase H digestion is highlighted by red dashed outline. (E) Representative images demonstrating neuronal phenotype of both cell lines, including synaptic vesicles (red outline) and neurofilaments (red arrows). (F-H) Different storage materials were observed in the CLN3 culture, including osmiophilic (red arrow) and fibrillar deposits (black arrow) (F), curvilinear structures (red arrow) (G) and multilamellar structures (red arrow) (H). (I) Quantification of autophagic vacuole (AV) area at DIV 42 (*n*=3 independent differentiated cultures, with data analyzed derived from 20-21 cells per cell line; linear mixed-effect model). (J) Representative fluorescence image showing DQ-BSA appearing as bright green puncta (inset) in the cytoplasm in CLN3-Cor neurons (left), further zoomed-in and indicated by red arrows (right). Scale bar: 15 μm. (K) DQ-BSA area in control and isogenic CLN3 neurons across different time points (*n*=3 independent differentiated cultures, with data analyzed derived from 1584-10,323 cells per cell line across all time points; zero-inflated beta regression model). All data, unless otherwise specified, are presented as group means±95% confidence interval; *P<0.05.

To explore lysosomal alterations in CLN3 neurons, we performed western blotting for detection of the lysosomal marker protein LAMP1. CLN3-Cor and genetically unrelated healthy control iPSC-derived neurons showed a single diffuse protein band of ∼90 kDa. In contrast, an additional band of ∼120 kDa was identified in CLN3 neurons (Fig. 3A). The total level of LAMP1 was higher in CLN3 neurons than in CLN3-Cor neurons at DIV 28 and DIV 42 (Fig. 3B). The ratio of LAMP1 120 kDa/90 kDa bands showed a decreasing trend from DIV 14 to DIV 42 (Fig. 3C). To identify the type of post-translational modification that may be related to the larger LAMP1 protein, protein extracts from control, CLN3 and CLN3-Cor neurons were treated with N-glycanase and endoglycosidase H. Western blot analysis showed a smaller band of ∼40 kDa in size after N-glycanase digestion (Fig. 3D). Meanwhile, western blot analysis of endoglycosidase H treatment showed a diffuse band of the larger-sized LAMP1 band in CLN3 neurons with full digestion of the 90 kDa LAMP1 form (Fig. 3D). This shows that the post-translational modification of the larger LAMP1 protein involves complex or hybrid N-linked glycosylation. As CLN3 mutation is associated with accumulation of subunit C of mitochondrial ATP synthase in cells, the subunit C levels in neuronal culture were examined through western blotting (Fig. S7A). Quantification of subunit C protein levels in the isogenic CLN3 neurons did not show differences through DIV 14, 28 and 42 (Fig. S7B).

In view of lysosomal alterations observed in LAMP1 western blotting, we further evaluated the ultrastructure of storage material in the isogenic CLN3 neurons through electron microscopy. Neurons from both genotypes have comparable neuronal phenotypes in terms of formation of synaptic vesicles and neurofilaments (Fig. 3E). In CLN3 cells, there were numerous autophagic vacuoles with a size range of 500-1000 nm, which were filled with heterogeneous storage material, including osmiophilic and floccular deposits (Fig. 3F), curvilinear structures (Fig. 3G) and multilamellar structures (Fig. 3H). The majority of storage material observed was in the form of multilamellar structures. However, ‘fingerprint’ deposits characteristic of CLN3 disease were not observed in any CLN3 sample. Quantification of the area of autophagic vacuoles revealed an increased autophagic vacuole area in CLN3 cells compared to CLN3-Cor cells (Fig. 3I).

Previous studies have demonstrated impairment of endocytosis in CLN3-deficient models (Fossale et al., 2004; Luiro et al., 2004). To investigate the effect of CLN3 mutation on endocytosis, we performed a DQ-bovine serum albumin (BSA) assay in the control and isogenic CLN3 cell lines, where formation of fluorescent DQ-BSA puncta indicates active endocytic trafficking process (Fig. 3J). Statistical tests comparing the DQ-BSA area in both cell lines revealed that although DQ-BSA area in the isogenic CLN3 cell lines was larger than that in the control cell line at DIV 28, DQ-BSA area in CLN3 neurons was significantly smaller than that in control and CLN3-Cor cell lines at DIV 42 (Fig. 3K).

Collectively, these results suggest that neurons with CLN3 mutation are associated with altered lysosome function and endocytosis compared to control and CLN3-Cor neurons. For a more in-depth characterization of the effects of CLN3 1 kb deletion mutation on an isogenic background, and to avoid potentially confounding effects from genetic variability from comparisons to unrelated control cell lines (Kilpinen et al., 2017), we explored the proteomic differences between CLN3 and CLN3-Cor neurons only.

### Alterations in pathways relating to protein homeostasis and trafficking, and axon guidance in CLN3 neurons

**Fig. 4.**
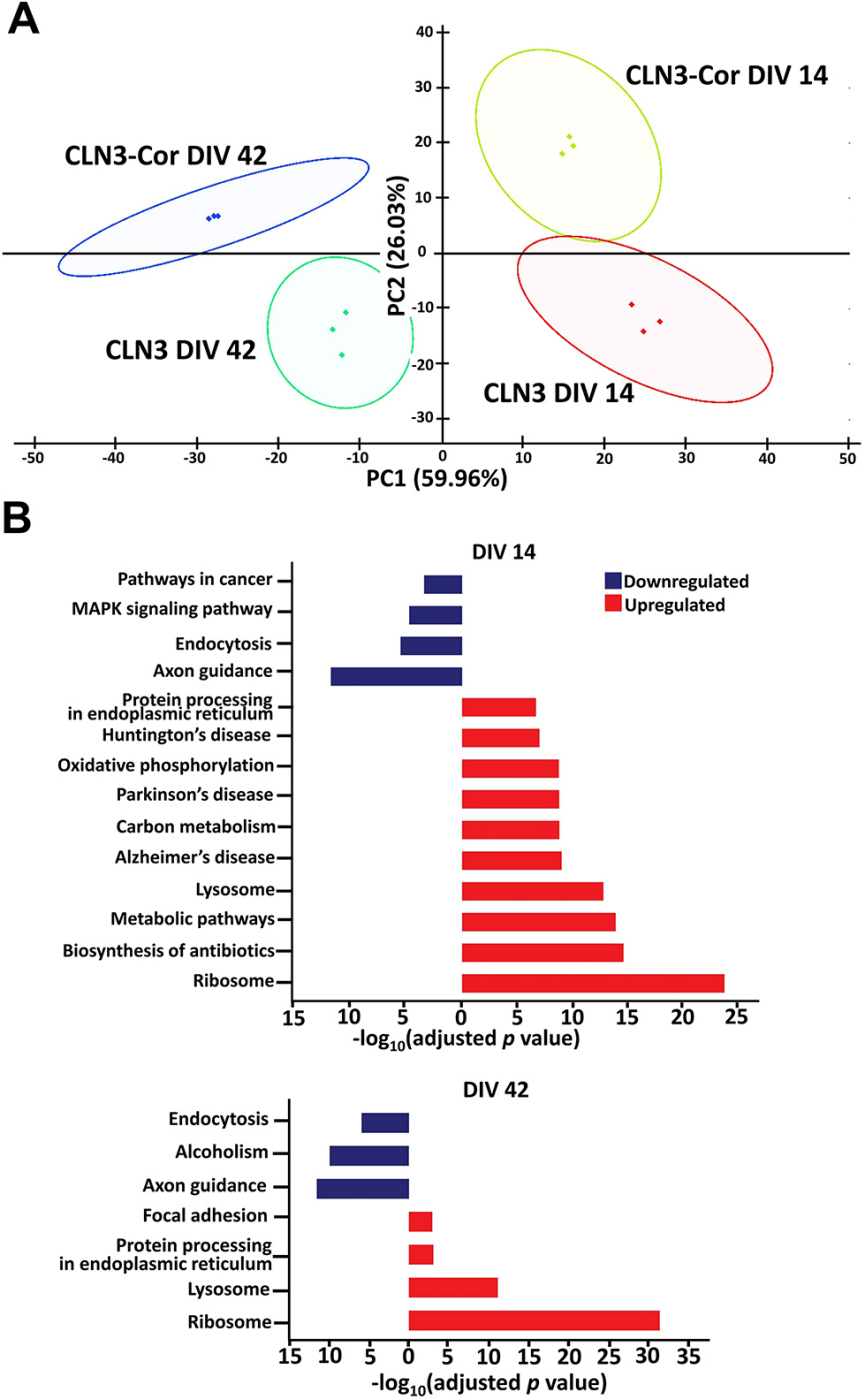
**Proteomic profile of isogenic CLN3 neurons.** (A) Principal component analysis plot of CLN3 and CLN3-Cor neurons at DIV 14 and 42. (B) Enriched Kyoto Encyclopedia of Genes and Genomes (KEGG) pathways in CLN3 neurons at DIV 14 and DIV 42 filtered based on thresholds [protein count ≥30 and −log_10_(Benjamini–Hochberg-adjusted *P* value ≥1.3)]; *n*=3 independent differentiated cultures per cell line per time point; one-way ANOVA.

CLN3 is associated with various cellular processes, and therefore CLN3 deficiency is likely to have broad effects. We explored global protein changes due to CLN3 mutation using quantitative proteomic analysis of protein extracts from isogenic CLN3 neurons at time points corresponding to early and late *in vitro* maturation of neurons. Differential expression analysis identified 2315 differentially expressed proteins at DIV 14 (1567 upregulated proteins and 748 downregulated proteins) and 1785 differentially expressed proteins at DIV 42 (1118 upregulated and 667 downregulated proteins). We investigated the effect of independent neuronal batches, genotype and DIV on the proteome using principal component (PC) analysis (PCA) (Fig. 4A). The PCA plot revealed clustering of biological replicates of CLN3 and CLN3-Cor neurons, but separation according to genotype and DIV. DIV explained 60% of the variation between samples (PC1), while genotype explained 26% of the variation (PC2). Bioinformatic analysis was then used to identify Kyoto Encyclopedia of Genes and Genomes (KEGG) pathways that were enriched in the sets of differentially abundant proteins. Significantly enriched pathways represented by the proteins detected at reduced levels in CLN3 neurons (DIV 14 and DIV 42) included endocytosis and axon guidance; the ribosomal and lysosomal pathways were over-represented among the proteins detected at higher levels in CLN3 neurons (Fig. 4B). The subsets of proteins related to ribosomes, lysosome, axon guidance and endocytosis pathways are represented in Fig. S8A-D according to their abundance ratios (CLN3/CLN3-Cor).

### Development of spontaneous firing activity in isogenic CLN3 cortical networks

**Fig. 5.**
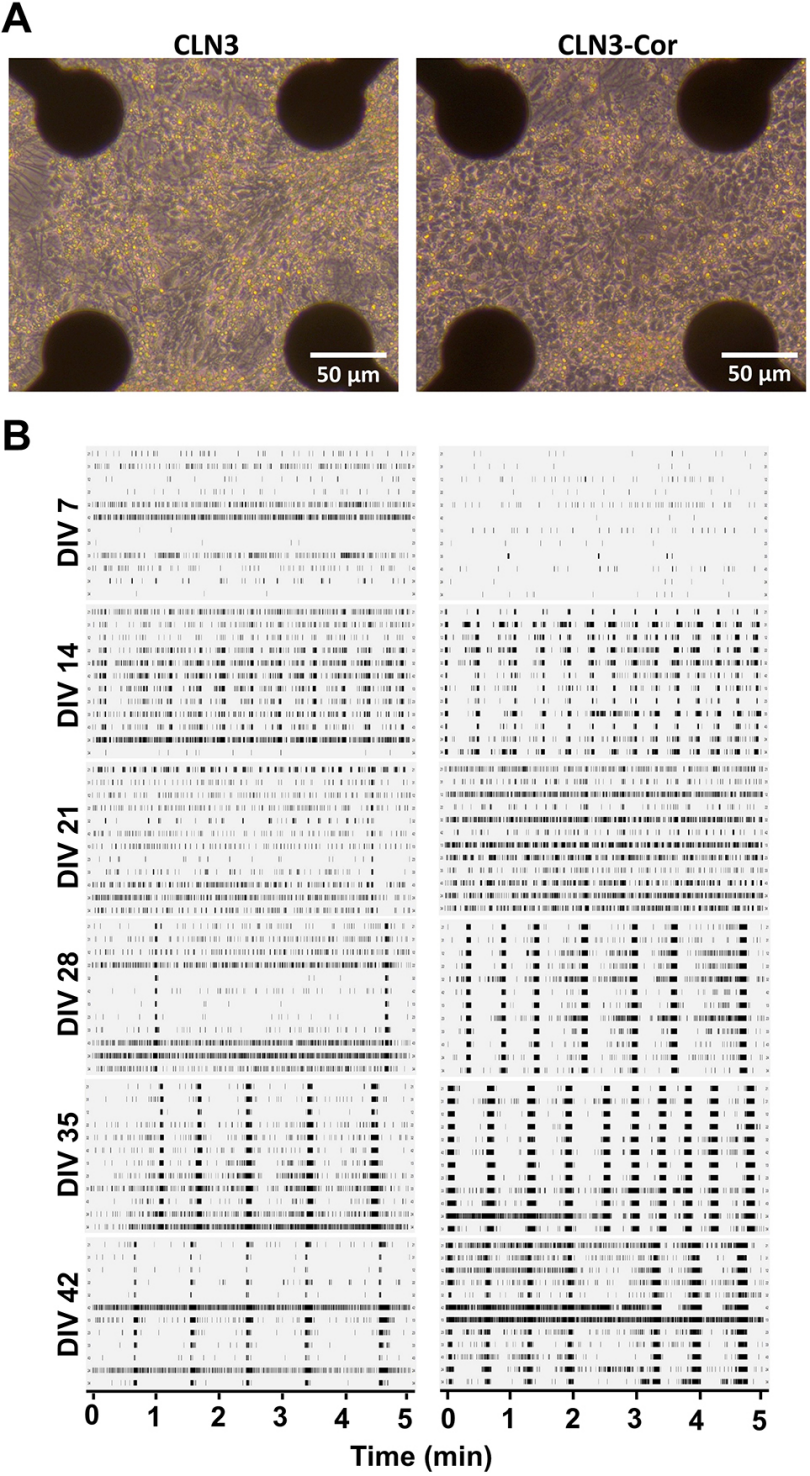
**Electrophysiological activity of isogenic CLN3 neurons on MEA.** (A) Representative images of CLN3 and CLN3-Cor neuronal cultures on MEA plates at DIV 10. (B) Representative raster plots of isogenic CLN3 neurons showing 5 min of electrophysiological activity across development from DIV 7 to DIV 42.

Proteomic analysis showed a downregulation of axon guidance-related protein expression in CLN3 neurons, suggesting reduced ability to form synapses. Relatedly, alterations to expression of ribosomal subunits have been shown to affect neurite arborization (Slomnicki et al., 2016), reflecting the altered morphology of CLN3 neurons compared to CLN3-Cor neurons (Fig. 2B). Therefore, we sought to determine whether alterations in neuronal activity patterns may be present in neurons with CLN3 mutation. To achieve this, we recorded the electrophysiological activity of iPSC-derived CLN3 and CLN3-Cor neurons using a multielectrode array (MEA), once a day from DIV 4 to DIV 42 (Fig. 2A). Differentiated neurons started growing new neurites immediately after seeding, followed by formation of interconnected circuits, where both cultures displayed an even cell distribution on the MEA plates (Fig. 5A) and fired spikes, bursts and network bursts (Fig. S9B) throughout the culture period (Fig. 5B).

**Fig. 6.**
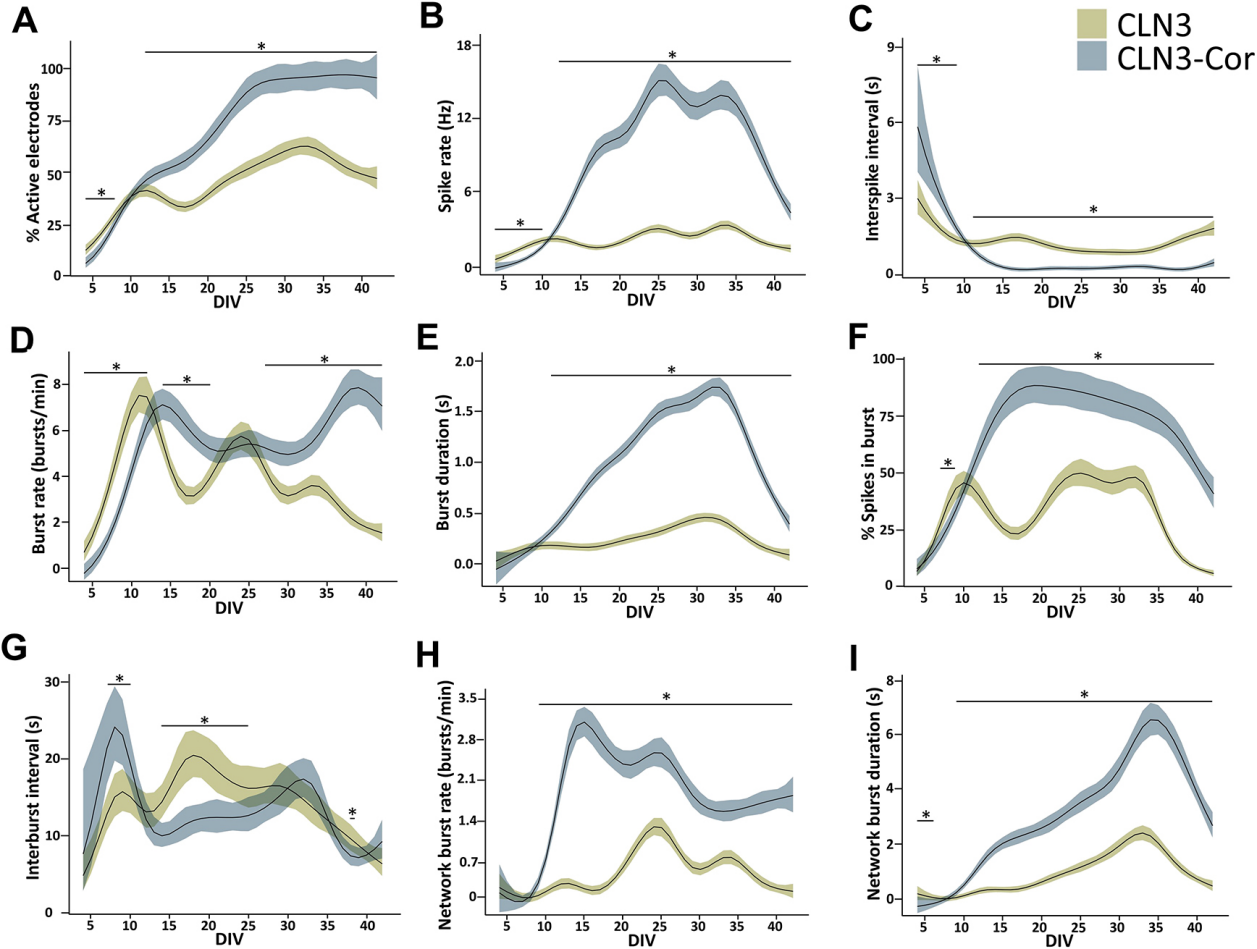
**Development of functional activity of isogenic CLN3 neurons on MEA.** (A-I) Graphs showing development of spike-related activities (A-C), burst-related activities (D-G) and network burst features (H-I) from DIV 4 to DIV 42. Data are presented as group means±95% confidence interval. *P<0.05; *n*=3 independently differentiated cultures, with data analyzed derived from 2-24 wells of 12 electrodes per cell line across all time points; generalized additive model.

The percentage of electrodes detecting activity (active electrodes) in both CLN3 and CLN3-Cor neurons peaked at DIV 32 (62.6%) and DIV 37 (97.1%), respectively (Fig. 6A). During DIV 4-8, the percentage of active electrodes was slightly higher in CLN3 neurons than in CLN3-Cor neurons (mean; CLN3, 20.8%; CLN3-Cor, 15.04%); however, from DIV 12 onward, CLN3 neurons had a lower percentage of active electrodes than in the CLN3-neurons (mean; CLN3, 48.97%; CLN3-Cor, 81.27%; Fig. 6A). The spike rate of CLN3 neurons increased from DIV 4 onward, peaked at DIV 12 (2.27 Hz) and then remained consistent toward DIV 42, while in CLN3-Cor neurons, spike rate increased from DIV 4 and peaked at DIV 25 (15.02 Hz), before declining toward DIV 42 (4.37 Hz) (Fig. 6B). We note that spike activities (and additional parameters discussed further below) in both cultures showed a decreasing trend at later stages of maturation (>DIV 35), which is similar to findings in other studies of iPSC-derived neurons and may be due to density of the cultures obscuring contact with the electrodes, or absence of glia in these cultures (Odawara et al., 2016; Tang et al., 2013; Hyvärinen et al., 2019).

CLN3 neurons from DIV 4 to DIV 10, had a higher spike rate than CLN3-Cor neurons (mean; CLN3, 1.37 Hz; CLN3-Cor, 0.56 Hz); however, from DIV 12 onward, CLN3 neurons fired less frequently than CLN3-Cor neurons (mean; CLN3, 2.36 Hz; CLN3-Cor, 10.53 Hz; Fig. 6B). In association with an increased spike rate, the interspike interval (ISI), the period of inactivity between spikes, in CLN3 neurons decreased between DIV 4 and DIV 12 (DIV 4, 3.02 s; DIV 12, 1.24 s) before plateauing toward DIV 42 (Fig. 6C). A similar trend was seen in CLN3-Cor neurons, in which the ISI declined rapidly from DIV 4 (5.85 s) to DIV 17 (0.21 s) before plateauing toward the end of the culture period (Fig. 6C). The higher spike rate, combined with the reduced ISI from DIV 12, indicates that CLN3-Cor neurons are more functionally active than CLN3 neurons.

### Characterization of burst and network burst profiles in isogenic CLN3 networks

In addition to early increases in spike rate (DIV 4-10), CLN3 neurons had higher burst rates than CLN3-Cor neurons (mean; CLN3, 4.45 bursts/min; CLN3-Cor, 2.52 bursts/min) during the early days of maturation (DIV 4-12; Fig. 6D). Burst rate peaked at 7.53 bursts/min (DIV 11) and 7.87 bursts/min (DIV 39) in CLN3 and CLN3-Cor neurons, respectively. As the culture matured further, CLN3-Cor neurons were bursting more frequently than CLN3 neurons during DIV 14-20 (mean; CLN3, 3.89 bursts/min; CLN3-Cor, 6.21 bursts/min) and DIV 27-42 (mean; CLN3, 2.9 bursts/min; CLN3-Cor, 6.29 bursts/min; Fig. 6D). Despite changes in burst rate, burst duration was similar between CLN3 and CLN3-Cor neurons during DIV 4-10 (mean; CLN3, 0.12 s; CLN3-Cor, 0.08 s); however, from DIV 11 onward, CLN3 neurons had shorter bursts than CLN3-Cor neurons (mean; CLN3, 0.27 s; CLN3-Cor, 1.12 s; Fig. 6E).

Additionally, the percentage of spikes transformed into bursts was considerably lower in CLN3 neurons than in the CLN3-Cor neurons at most cultured time points, indicating that the spiking activity of CLN3-Cor neurons led to bursts more often than in CLN3 neurons (Fig. 6F). During DIV 7-9, CLN3 neurons had a higher percentage of spikes in bursts (mean, 36.06%) than CLN3-Cor neurons (mean, 27.20%); however, from DIV 12 onward, CLN3-Cor neurons (mean, 75.98%) had a higher percentage of spikes in bursts than CLN3 neurons (mean, 33.38%), indicating increased activity in CLN3-Cor neurons (Fig. 6F). The percentage of spikes in bursts peaked at 50.26% (DIV 25) and 88.69% (DIV 19) in CLN3 and CLN3-Cor neurons, respectively, followed by a general decline in both lines toward DIV 42 (CLN3, 6.29%; CLN3-Cor, 41.21%; Fig. 6F).

The time between bursts, known as the interburst interval (IBI), of CLN3 neurons peaked at DIV 18 (20.45 s) before decreasing toward DIV 42 (6.28 s; Fig. 6G). In CLN3-Cor neurons, the IBI increased from DIV 4 (7.56 s) to DIV 8 (24.14 s), followed by a general declining trend toward DIV 42 (9.19 s; Fig. 6G). In association with an increased burst rate, the IBI was shorter in CLN3 neurons than in CLN3-Cor neurons (mean; CLN3, 14.61 s; CLN3-Cor, 21.96 s) during DIV 7-10 (Fig. 6G). As the culture matured during DIV 14-25, longer IBI (mean; CLN3, 17.85 s; CLN3-Cor, 11.73 s) occurred concurrently with the lower burst rate in CLN3 neurons than in CLN3-Cor neurons (Fig. 6G).

In CLN3 neurons, the network burst rate, defined as the rate of synchronous network activity between ≥3 electrodes per well per minute, peaked at DIV 24 (1.24 bursts/min) and subsequently showed a general declining trend toward DIV 42 (0.10 bursts/min; Fig. 6H). CLN3-Cor neurons generated network bursts more frequently than CLN3 neurons from DIV 9 onward (mean; CLN3, 0.48 bursts/min; CLN3-Cor, 2.00 bursts/min), where the CLN3-Cor neuronal network burst rate peaked at DIV 15 (3.09 bursts/min) before declining toward DIV 42 (1.79 bursts/min; Fig. 6H). Although CLN3 and CLN3-Cor neurons demonstrated an increasing trend in network burst duration during DIV 4-34 [peak; CLN3, 2.42 s (DIV 33); CLN3-Cor, 6.52 s (DIV 34)], network burst duration was longer in CLN3-Cor neurons than in CLN3 neurons from DIV 9 to DIV 42 (mean; CLN3, 1.04 s; CLN3-Cor, 3.48 s; Fig. 6I). The higher burst and network burst activity in CLN3-Cor neurons for most of the culture period indicate that CLN3-Cor cultures fire more often, for longer, and in a more coordinated manner than CLN3 cultures.

## DISCUSSION

Our isogenic, patient-specific iPSC-derived neuronal cell model provides new insight into protein homeostasis and electrophysiological alterations in CLN3 disease that point to previously under-recognized mechanisms that may contribute to neurodegeneration. More broadly, our data demonstrate the utility of iPSCs for modeling CLN3 disease, and imply that such models will be a powerful tool for identifying and testing new therapies for this disease.

We observed hyperglycosylated LAMP1 in CLN3 neurons that was not apparent in CLN3-Cor or genetically unrelated healthy control iPSC-derived neurons. To our knowledge, our study is the first to report the occurrence of LAMP1 hyperglycosylation in a CLN3 disease model. However, a similar finding has been observed in *Npc1*^−/−^ mice, a model of Niemann-Pick disease (NPC) type C, and which has been associated with Purkinje neuron loss (Cawley et al., 2020). The hyperglycosylation of LAMP1 in our CLN3 neuronal model occurred concurrently with altered levels of NPC1 and NPC2, as observed in our proteomics study. NPC1 and NPC2 are lysosomal glycoproteins, which, when mutated, are a cause of NPC disease and act in egress of cholesterol from lysosomes to the cytoplasm (Carstea et al., 1997; Naureckiene et al., 2000). Hyperglycosylated LAMP1 has been shown to impair transportation of cholesterol from the lysosome to the endoplasmic reticulum in NPC1 protein-deficient cells (Li et al., 2015). Therefore, LAMP1 hyperglycosylation and increased levels of NPC1 and NPC2 in CLN3 neurons could be associated with accumulation of cholesterol in the lysosomes (Li et al., 2015). This distinct hyperglycosylated form of LAMP1 was observed predominantly in CLN3 neurons at early time points, but to a lesser extent at the later time point, which could be a compensatory response to accumulated contents (for example, cholesterol) in the lysosome.

Ultrastructural studies of iPSC-derived neurons also implicate lysosomal dysfunction in this model of CLN3 disease, and further point to accumulation of lipid species. For example, osmiophilic deposits are suggestive of lipid storage and are also observed in models of *CLN1* (encoding PPT1) mutation. CLN1 deficiency and other lipid storage disorders such as Gaucher disease are associated with the accumulation of saposin A and D, which degrade sphingolipids (Tyynelä et al., 1995). The accumulation of multilamellar membranous whorls in the storage contents of CLN3 neurons is also suggestive of disruption in lipid degradation (Parkinson-Lawrence et al., 2010). In contrast, the curvilinear storage materials observed in CLN3 neurons in this model are the dominant ultrastructure of storage bodies in CLN2 disease. Curvilinear storage materials comprise mainly proteins and are associated with subunit C of mitochondrial ATP synthase, a highly hydrophobic and major accumulating protein in CLN2 mutation (Goebel et al., 2001; Palmer et al., 1992). The presentation of heterogeneous storage materials in CLN3 neurons highlights the possibility of deficit in degradation of multiple types of cellular wastes comprising protein, lipid and glycan. The appearance of heterogeneous storage materials observed in CLN3 neurons, rather than the predominant fingerprint profile observed in typical CLN3 mutations, suggests that this may be a CLN3 variant or cell line-specific ultrastructural phenotype, but which requires confirmation using other CLN3 models.

Our proteomic data identified several pathways relevant to protein homeostasis and implicate CLN3 as having broader effects on the proteome beyond substrate accumulation in lysosomes. Our finding of upregulation of lysosome-related proteins at DIV 14 (49 proteins) and DIV 42 (40 proteins) is in accordance with previous research, which demonstrated significant elevation of lysosomal proteins SCARB2, HEXB and TPP1 in CLN3 mouse brain (Sleat et al., 2019), HEXA and LAMP1 in urine of CLN3 patients (Iwan et al., 2021), PPT1 and GLA in lysosomal fractions of CLN3*^Δex7/8^* mouse cerebellar cells (Schmidtke et al., 2019), and PPT1, TPP1, NAGA and NAGLU in CLN3 brains following autopsy (Sleat et al., 2017). The changes in lysosomal protein abundance may be a compensatory cellular response to the accumulation of various substrates within the lysosome. Among the lysosomal proteins that were upregulated with higher fold change at DIV 42 compared to DIV 14 were CTSC, TPP1, NAGA and GM2A. The elevation of these proteins in our study indicates possible increase in the catalysis of substrates such as gangliosides by GM2A, HEXA and HEXB (Cordeiro et al., 2000), protein by TPP1 and CTSC (Kim et al., 2008), and glycoprotein by NAGA (Michalski and Klein, 1999) to prevent the accumulation of these substrates. Notably, the accumulation of GM2A within lysosomes of neurons which occurs in Tay–Sachs disease, another lysosomal storage disorder, has led to neuronal death (Drozd et al., 2015). A separate study in *Cln3^Δex7/8^* mice has also reported accumulation of GM3 gangliosides due to altered expression of ganglioside-metabolizing enzymes (Somogyi et al., 2018). TPP1 has been suggested to be involved in the degradation of subunit C of mitochondrial ATP synthase (Ezaki et al., 1999). Therefore, elevation of TPP1 activity, which has been described in CLN3 mouse brain (Sleat et al., 1998) and CLN3 patient brains (Junaid and Pullarkat, 1999; Sleat et al., 1998), may be important to prevent accumulation of subunit C.

A second pathway associated with protein homeostasis identified in our proteomic studies is related to ribosomes. This was the top-ranked pathway at both time points examined, with many proteins that form the large and small ribosomal subunits being more highly expressed in CLN3 neurons. CLN3 has been previously linked to ribosomes, with yeast models demonstrating a genetic interaction with several genes encoding ribosomal proteins (Minnis et al., 2021), and protein–protein interaction with SBDS, which is involved in ribosome biogenesis (Vitiello et al., 2010). More recently, CLN3 ablation has been reported to cause reduced translation in HeLa cell models (Relton et al., 2022 preprint). Pathological gene variants and altered expression level of the translational machinery including initiation factors, elongation factors, ribosomal subunits and tRNA synthetase genes have been implicated in many other neurological disorders (for review, see Skariah and Todd, 2021), but precisely how disruption of ribosomes contributes to neurodegeneration in CLN3 disease is not yet known.

In addition to pathways related to protein homeostasis, our proteomic analysis identified downregulation of endocytosis-related proteins, a finding supported by data from our DQ-BSA experiment, indicating that CLN3 mutation may have a minor negative effect on endocytosis. Impaired endocytosis has been reported in CLN3 patient fibroblasts, CLN3-deficient yeast (Codlin et al., 2008), CLN3 mouse cerebellar cells (Fossale et al., 2004) and CLN3 endothelial cells (Tecedor et al., 2013). In CLN3 neurons, proteins related to clathrin-mediated endocytosis, which is essential for synaptic plasticity, neurotransmission (Kononenko et al., 2014) and axonal pathfinding during development (Yap and Winckler, 2015), were downregulated. Interestingly, mouse models of CLN3 disease reveal increased cell-surface expression of the GluA1 and GluA2 subunits of the AMPA receptor, leading to increased sensitivity to the receptor agonists AMPA and kainic acid (Kovács and Pearce, 2008; Kovács et al., 2006, 2015). In this regard, the AMPA receptor is endocytosed via a clathrin-mediated mechanism (Lu et al., 2007), and it would be interesting to test AMPA and kainic acid sensitivity in our isogenic iPSC model.

Our proteomic findings suggest that CLN3 deficiency alters protein homeostasis by affecting translation, trafficking and degradation, and that disruption of these pathways may negatively affect synaptic transmission or axonal growth. Therefore, we compared the functional activity of neuronal maturation over time in our isogenic CLN3 models using MEA by analyzing selected features of spikes, bursts and network events. Although spikes and bursts reflect the overall activity of the neuronal network, network burst events demonstrate the synchronicity in spikes and bursts within the neuronal network (Mossink et al., 2021). The MEA findings showed that CLN3-Cor and CLN3 neuronal networks fired spikes and bursts, and, upon maturation, increased connectivity and synchronicity was observed within network bursts. However, the level and time scale of acquiring activity varied between cell lines, such that CLN3-Cor neurons fire more frequently, for longer, and in a more coordinated manner than CLN3 neurons. Network bursts play an important role in nervous system development and maturation, and are strongly correlated with the strengthening of both glutamatergic and GABAergic synapses (van Pelt et al., 2005; Ichikawa et al., 1993; Ito et al., 2013). Our proteomic data revealed that several proteins involved in the glutamatergic synapse pathway in the CLN3 neurons were expressed at levels lower than those in CLN3-Cor neurons at both time points examined. These include SLC1A2, GRIK3, PPP3CB, GRM7, DLG4, GNAQ, GNAS, SLC17A6, PLCB1, PRKACA and PRKACB. At DIV 42, proteins involved in the GABAergic synapse pathway – including GABRB3, PRKCB, SRC, PLCL1, GABRA3, ADCY8, GPHN, GNAl1, GLS, GNAO1, GNG2, GNB4, PRKACA and PRKACB – were expressed at lower levels in CLN3 neurons than in CLN3-Cor neurons. Additionally, during the later stage of maturation (DIV 28), CLN3 neurons had lower expression of several mRNAs encoding glutamatergic (*GRIA2*, *SLC1A2*) and GABAergic (*GAD2*) neurons and synaptic proteins (*DLG4*). Collectively, these data point to CLN3 mutation causing molecular alterations at synapses that decrease neuronal network activity, and which could represent an early stage of neuronal dysfunction underlying CLN3-related disease phenotypes.

Alternatively, the reduced activity of neuronal networks in CLN3 neuron cultures could be related to neurodevelopmental aspects of CLN3 mutation, as has been suggested for Cln3 (and Cln2 and Cln5) in mouse neuronal development (Fabritius et al., 2014; Minye et al., 2016). Our proteomic data provide additional support for neurodevelopmental alterations in CLN3 neurons, showing the downregulation of axon guidance-related proteins in CLN3 neurons, including several important classes of axon guidance molecules and their receptors. These include, for example, NTNG1 guidance cue, which binds to the transcription activator DCC (Russell and Bashaw, 2018), L1CAM (cell adhesion molecule), which is essential for axonal elongation and dendritic arborization (Patzke et al., 2016), ROBO1 and ROBO2 receptors, which guide major forebrain axonal projections (López-Bendito et al., 2007), and SEMA4D, a semaphorin that repels axons (Raissi et al., 2013). Central nervous system embryonic development requires neurons to extend axons to distant targets and to develop functional circuits (Pasterkamp and Burk, 2021). Disruption to axon guidance and synapse formation could therefore affect neuronal differentiation and maturation, potentially explaining the lower levels of firing and network burst activity in the CLN3 neurons. Of note, other human iPSC-based models of CLN3 disease have reported difficulty in differentiation of *CLN3*^△ex7/8/△ex7/8^ neural progenitor cells (Lojewski et al., 2014), and severe developmental failure of some, but not all, CLN3^Q352X^ organoids upon initiation of differentiation has also been described (Gomez-Giro et al., 2019).

Intriguingly, the top-ranked pathways related to protein homeostasis can be linked to neurite growth and synaptic transmission. Alterations to lysosomal proteins may also have an impact on axon growth, where inhibition of lysosome transport to the distal axon alters the size and dynamics of growth cone, which could be related to disruption of lysosome-mediated delivery of signaling and adhesion molecules (Farías et al., 2017) or local degradation of cargos in axons to maintain axon homeostasis (Manzoli et al., 2021). We note that if, as this study and others have suggested, CLN3 mutation decreases protein translation and trafficking, then this is also likely to impact on the functioning of the axon guidance pathway, particularly given the highly arborised morphology of neurons, which creates a need for efficient protein trafficking to establish and maintain synaptic connectivity. Indeed, localized translation occurs within axons (Dalla Costa et al., 2021), and this mechanism may couple two or more of the pathways we have identified in our proteomic analysis. Defining the axonal proteome in CLN3 disease would be an important next step in understanding how CLN3 regulates axonal homeostasis.

Although we have undertaken experiments to demonstrate the integrity of our iPSC lines, our data need to be considered with care and require verification in other models. This could include additional pairs of isogenic cell lines, derived from distinct patients carrying the 966 bp deletion, or through engineering of this deletion into iPSCs from unaffected individuals as others have done for other mutations (Scotto Rosato et al., 2022; Gomez-Giro et al., 2019). There is also significant genetic heterogeneity in CLN3 disease (Gardner and Mole, 2021), and different pathological variants may affect CLN3 in different ways. For example, the E295K and other missense variants are trafficked to lysosomes, whereas the 966 bp deletion and other frameshifting and nonsense mutations cause CLN3 to be retained within the endoplasmic reticulum (Haskell et al., 2000). Thus, our findings may not be immediately applicable across the spectrum of CLN3 disease, and there remains a need to completely understand how the many disease-causing variants affect CLN3 function. Last, we have only reported data from one affected brain cell type. Although others have studied, for example, retinal cell types (Tang et al., 2021), ideally different brain cell types (e.g. astrocytes and microglia) that are affected by CLN3 mutation (Parviainen et al., 2017) and organs such as the eye should be studied using the same iPSC lines to enable direct comparison of how CLN3 variants affect each of these tissues. We have now initiated iPSC-focused projects aimed at addressing many of these questions.

Findings from this study further our understanding of cellular alterations in CLN3 disease, especially the lysosomal pathway and neurodevelopmental aspects, caused by a compound heterozygous genotype associated with an atypical, protracted phenotype (Munroe et al., 1997). Our study of phenotypes in isogenic neurons could potentially aid in lead-molecule efficacy testing or drug discovery screens to halt disease progression before neurodegeneration occurs, and which may be applicable to other CLN3 genotypes in addition to that studied here. For example, miglustat, an iminosugar that inhibits the synthesis of glycosphingolipid, is subject to a clinical trial for efficacy in the treatment of CLN3 disease (NCT05174039). Future studies using iPSC-derived neuronal models that capture a diversity of CLN3 genotypes will help to uncover these drug targets.

## MATERIALS AND METHODS

### Ethics and biological safety approvals

The use of human biological material for the research purpose of this study was approved by the Human Research Ethics Committee of Tasmania (H0014124) and the Royal Victorian Eye and Ear Hospital (11/1031) following protocols that involved obtaining informed consent from participants or their guardian, and was conducted according to the principles expressed in the Declaration of Helsinki. Work involving genetic manipulation was approved by the University of Tasmania Institutional Biosafety Committee.

### Human fibroblast culture

Primary fibroblasts, MBE 02873 (*CLN3* Δ 966 bp and E295K, hereafter designated CLN3 line), were established from a patient skin biopsy as described (Neavin et al., 2021). Fibroblasts were grown in medium containing 88% Dulbecco's modified Eagle medium (DMEM) with high glucose (Gibco) supplemented with 10% fetal bovine serum (Gibco), 1% non-essential amino acids (Gibco) and 1% antibiotic-antimycotic (Gibco) in T75 flasks.

### Generation of iPSCs from CLN3 patient fibroblasts

CLN3 fibroblasts were reprogrammed using an Epi5 Episomal iPSC reprogramming kit (Invitrogen). The episomal vectors encoding *OCT4*, *SOX2*, *LIN28* (*LIN28A*), *KLF4* and *L-MY*C (*MYCL*) were transfected using Lipofectamine 3000 reagent (Invitrogen). Live surface marker staining with Anti-TRA-1-60 488 Live Cell Stain (Miltenyi Biotec) was performed at day 21 post-lipofection to identify undifferentiated iPSC colonies in the culture. Newly reprogrammed iPSCs were maintained in mTeSR Plus Medium (Stemcell Technologies) on Matrigel-coated plates. The TOB00220 iPSC line, herein used as a healthy control cell line, was published previously (Crombie et al., 2017). All cell lines were routinely tested for mycoplasma using protocols we have described previously (Wang et al., 2021) and were negative.

### Detection of episomal vector

To detect the presence of episomal vectors in reprogrammed iPSCs, endpoint PCR was performed on genomic DNA extracted from the iPSCs using EBNA-1 primers (Table S2), which identified all five episomal plasmids in the Epi5 reprogramming kit.

### Assessment of iPSC pluripotency, differentiation potential and cell line identification

iPSCs were stained with primary antibodies rabbit anti-NANOG (1:200), rabbit anti-OCT4 (1:200), rabbit anti-PAX6 (1:200), mouse anti-TRA-1-60 (1:200), mouse anti-TRA-1-81 (1:200) and mouse anti-SSEA4 (1:200) (all from Cell Signaling Technology, StemLight Pluripotency Antibody Kit, 9656) to assess for pluripotency. To further assess pluripotency and trilineage differentiation potential of iPSCs, EBs were formed. iPSCs at 80% confluency were dissociated with ReLeSR (Stemcell Technologies). Cell aggregates were transferred into ultra-low attachment six-well plates in EB medium consisting of 20% knockout serum replacement (Gibco), 79% DMEM/F-12, GlutaMAX supplement (Gibco), 1% non-essential amino acids and 0.1 mM 2-mercaptoethanol (Sigma-Aldrich) for EB formation. Medium was refreshed every 2 days. On day 4, EBs were plated onto Matrigel-coated wells for further differentiation in EB medium for 10 days. Expression of pluripotency and germ layer genes of EBs on day 14 of differentiation was examined using a TaqMan hPSC Scorecard Panel (Thermo Fisher Scientifc) following the manufacturer's instructions. Data analysis was done using web-based hPSC Scorecard Analysis software (https://www.thermofisher.com/au/en/home/life-science/stem-cell-research/taqman-hpsc-scorecard-panel/scorecard-software.html). STR profiling and analysis of CLN3 fibroblasts and iPSCs (parental and isogenic corrected line) were carried out by Australian Genome Research Facility (Melbourne, Australia).

### Virtual karyotyping analysis

Copy number variation (CNV) analysis of parental fibroblasts and iPSCs was performed using an Illumina HumanCytoSNP-12 beadchip array (Illumina). B allele frequency and log R ratio of each single-nucleotide polymorphism (SNP) marker were collected from GenomeStudio (Illumina) and analyzed with PennCNV (Wang et al., 2007) and QuantiSNP (Colella et al., 2007) with default parameter settings. Genomic regions with CNV calls having at least 20 contiguous SNPs or genomic regions with SNPs spanning at least 1 Mb generated by both PennCNV and QuantiSNP were then visualized using GenomeStudio to confirm the absence of chromosomal aberration (Neavin et al., 2021).

### Design of crRNA and donor plasmid

crRNA, CAAGGTAGGGACTTGAAGGA, which targets the breakpoint sequence of CLN3 966 bp deletion, was designed using the Benchling platform (www.benchling.com). A ∼3.7 kbp donor repair construct was cloned into pUC57 vector at the EcoRV site by GenScript (Nanjing, China). The donor plasmid consists of the corrected *CLN3* sequence with LoxP-flanked puromycin-selection cassette flanked by 801-911 bp of homologous sequence.

### CRISPR/Cas9-mediated correction of 966 bp deletion

Editing of CLN3 iPSCs to correct 966 bp deletion was done essentially as described (Wang et al., 2021). A single-cell suspension (800,000 cells) of CLN3 iPSCs was nucleofected with Cas9-sgRNA ribonucleoprotein (IDT) and 966 bp donor plasmid using Amaxa 4D Nucleofector (Lonza) with program CB 150. iPSCs were treated with puromycin 72 h after CRISPR/Cas9 electroporation when cells had reached ∼80% confluency. Cells were cultured in 0.125 µg/ml (half the kill dose) puromycin in mTeSR Plus Medium for 3 days, with daily medium replacement. Cells were then cultured in 0.25 µg/ml puromycin in mTeSR Plus Medium for another 3 days. When distinct colonies appeared large enough for picking (∼200 µm), they were isolated for further expansion and genotyping. DNA extract from edited clones was amplified through PCR reaction using CLN3 F1 and CLN3 R1 primers that span the 966 bp deletion region. Successfully edited cells, as confirmed by PCR, were treated with Cre recombinase gesicles (Takara Bio) with 6 µg/ml Polybrene (Sigma-Aldrich) in mTeSR Plus Medium to remove the puromycin-resistance cassette. Successful Cre-Lox recombination was confirmed by PCR using CLN3 F1 and R1 primers. Sanger sequencing of cDNA of the edited clone was performed using primers CLN3_SS_cdna_F1 and CLN3_SS_cdna_R1. All primers used are listed in Table S2.

### Off-target analysis

The top ten predicted off-target sites including all predicted off-target sites within exonic regions were determined using Benchling (www.benchling.com). Sanger sequencing was performed to sequence the 300-400 bp sequences surrounding the off-target sites in both edited and native unedited cell lines. Indels were analyzed with Synthego ICE analysis tool (https://ice.synthego.com/#/).

### Neural differentiation

Neural induction and differentiation were done essentially as described (Talbot et al., 2022). When the iPSCs were 80% confluent, cells were dissociated into small clumps using Accutase and seeded at a density of 3×10^5^ cells/well in poly-l-ornithine (PLO; Sigma-Aldrich)/laminin (Gibco)-coated six-well plates. Cells were cultured in mTeSR Plus Medium supplemented with 10 µM Rho-associated protein kinase (ROCK) inhibitor (Stemcell Technologies). After overnight incubation, the spent medium was replaced by Neural Induction Medium containing 98% Neurobasal Medium (Gibco) and 2% Neural Induction Supplement (Gibco), which was subsequently refreshed every other day. On day 7 of culture, primitive NSCs were dissociated with Accutase and seeded at a density of 8×10^5^ cells/well in a PLO/laminin-coated plate. Cells were cultured in Neural Expansion Medium (NEM) consisting of 49% Neurobasal Medium, 49% Advanced DMEM (Gibco) and 2% Neural Induction Supplement (Gibco) pre-treated with 5 µM ROCK inhibitor. NEM without ROCK inhibitor was changed every other day until cells were confluent.

For differentiation into mature neurons, NSCs at passage 4 were plated onto PLO/laminin-coated six-well plates. When cells were 75-90% confluent, NEM was replaced with neuronal differentiation medium consisting of 95% Neurobasal Plus Medium (Gibco) supplemented with 2% B-27 Plus (Gibco), 1% GlutaMAX (Gibco), 1% CultureOne (Gibco) and 1% antibiotic-antimycotic. The next day, half-medium exchange was performed. On day 4, cells were dissociated with Accutase and seeded into PLO/laminin-coated six-well plates containing neuronal maturation medium (95% Neurobasal Plus Medium, 2% B-27 Plus, 1% GlutaMAX, 1% CultureOne, 1% antibiotic-antimycotic supplemented with 10 ng/ml BDNF (Stemcell Technologies), 10 ng/ml GDNF (Stemcell Technologies), 0.25 mM db-cAMP (Sigma-Aldrich) and 0.2 mM L-ascorbic acid (Sigma-Aldrich). Cells were cultured for 42 days in neuronal maturation medium. Medium was replaced with fresh pre-warmed medium every 2-3 days with half-volume change.

### Immunofluorescence of NSC and neurons

NSCs (10,000 cells/well) and neurons (25,000 cells/well) were seeded in 96-well tissue culture-treated plates. After an appropriate time in culture, cells were then fixed with 4% paraformaldehyde (PFA; Sigma-Aldrich) in phosphate-buffered saline (PBS; 0.01 M) for 20 min at room temperature on an orbital shaker. Cells were washed with PBS (3×10 min) and permeabilized with 0.1% Triton X-100 (Sigma-Aldrich) in PBS for 10 min. Fixed cells were incubated for 1 h in blocking solution (5% fetal bovine serum in PBS) followed by incubation with primary antibodies. NSCs were characterized by the expression of NES (Stemcell Technologies, 69001; 1:1000) and PAX6 (Stemcell Technologies, 69001; 1:500). Neurons were characterized by the expression of MAP2 (Abcam, ab5392; 1:3000) and TAU (Dako, A0024; 1:5000). After incubation with primary antibodies, cells were washed with PBS (3×5 min). Cells were incubated with species-appropriate secondary antibodies conjugated to Alexa Fluor fluorochromes (Invitrogen, A32931 and A31572; 1:1000) at room temperature for 2 h. Secondary antibodies were aspirated, and cells were incubated with 5 µg/ml 4′,6-diamidino-2-phenylindole (DAPI; Merck) in PBS for 20 min. Cells were washed with PBS three times, and 0.02% w/v sodium azide (Merck) in PBS was added before imaging on a Celldiscoverer 7 (Zeiss).

### RNA extraction and RT-qPCR

RNA was extracted from iPSCs, NSCs and neurons at DIV 7, 14, 28 and 42 using an RNAeasy Plus Mini Kit (Qiagen). cDNA was synthesized using 1 µg RNA per sample according to the manufacturer's instructions (Omniscript RT kit, Qiagen). Taqman assay probes (Thermo Fisher Scientific) used for RT-qPCR are listed in Table S3.

### Western blotting

Proteins from neuronal culture at DIV 14, 28 and 42 grown in a 12-well plate were extracted using 50 µl ice-cold RIPA lysis buffer (Abcam) added with protease inhibitor (Roche) and phosphatase inhibitor (Roche). Protein concentration was determined by performing Pierce BCA Protein assay according to the manufacturer's instructions (Thermo Fisher Scientific). After western blotting, membranes were incubated with anti-LAMP1 (Cell Signaling Technology, D2D11; 1:4000), anti-subunit C of ATP synthase (Abcam, ab181243; 1:4000) or the loading control, anti-GAPDH (Merck, AB2302; 1:120,000). Densitometric analysis of protein bands was performed using Image Studio Lite Version 5.2 to derive band intensities, which were then normalized to GAPDH. Raw western blot images are provided in Fig. S10.

### Deglycosylation of LAMP1

Proteins from neuronal culture at DIV 14 were extracted as described above and treated with and without N-glycanase or endoglycosidase H according to the manufacturer's instructions (New England Biolabs). The lysates were analyzed with western blotting using anti-LAMP1 (1:4000) antibody.

### Transmission electron microscopy

Neuronal culture in confluent 12-well plates was fixed directly with gentle agitation for 15 min at room temperature with a fixative containing 2.5% glutaraldehyde (Electron Microscopy Sciences), 2% PFA (Electron Microscopy Sciences), 0.025% calcium chloride (Sigma-Aldrich) in a 0.1 M sodium cacodylate (Electron Microscopy Sciences) buffer pH 7.4. Cells were washed (3×10 min) with cacodylate buffer. Cells were scraped gently into a microfuge tube and post-fixed in 1% osmium tetroxide (Electron Microscopy Sciences) for 30 min to 1 h at 4°C. Cells were washed with Milli-Q water (3×10 min), followed by overnight staining with 0.5% uranyl acetate (Electron Microscopy Sciences) at 4°C. The next day, uranyl acetate was activated through heating at 50°C for 1 h. Cells were washed with Milli-Q water (3×10 min) and dehydrated in graded ethanol solutions and propylene oxide on ice in the order 50%, 70%, 80%, 90%, 100% (5 min each), propylene oxide (3×5 min), and infiltrated overnight with propylene oxide: resin (1:1). The next day, cells were embedded in resin and polymerized for 2 days at 60°C. After polymerization, thin sections (70 nm) from resin blocks were cut and stained with Uranyless solution (ProSciTech) for 7 min followed by lead citrate (Sigma-Aldrich) for 7 min. Images were captured with a Hitachi 7700 transmission electron microscope with a LaB6 filament, at 80 kV in high-contrast mode.

The area of late degradative autophagic vacuoles having one limiting membrane with cytoplasmic materials (Lojewski et al., 2014) was quantitated in 20-21 cells derived from three independent differentiated cultures per genotype. Regions of interest were outlined using ImageJ to quantitate the autophagic vacuole area.

### DQ-BSA assay

Cells were seeded at a density of 25,000 cells/well in a black flat-bottom tissue culture-treated 96-well plate. On the day of the experiment, cells were rinsed once with pre-warmed DPBS^−/−^ (Gibco) then incubated with 10 µg/ml DQ Green BSA (Invitrogen), which was diluted in medium for 6 h at 37°C, 5% CO_2_. After incubation, DQ Green BSA was removed, and cells were fixed with 4% PFA for 20 min at room temperature. Cells were washed with PBS (3×10 min) and incubated with 5 µg/ml DAPI for 10 min. Cells were imaged with a Celldiscoverer 7 using At495 (495/527 nm) and DAPI channels. DQ-BSA puncta (diameter, 3-9 pixels) in cell bodies were segmented, and area of DQ-BSA puncta, normalized to cell area, was quantified. Image analysis was performed using CellProfiler 4.2.1. Numbers of cells quantified were as follows: DIV 14 (control, 8503 cells; CLN3, 1584 cells; CLN3-Cor, 9266 cells), DIV 28 (control, 4941 cells; CLN3, 1816 cells; CLN3-Cor, 7073 cells), DIV 42 (control, 5489 cells; CLN3, 2033 cells; CLN3-Cor, 10323 cells).

### Protein preparation for mass spectrometry

Proteins were harvested from CLN3 and CLN3-Cor neurons (*n*=3 independent differentiated cultures per cell line), which had been cultured in six-well plates. Protein was extracted using 100 µl lysis buffer (7 M urea (Sigma-Aldrich), 2 M thiourea (Sigma-Aldrich) and 30 mM Trizma base (Sigma-Aldrich) containing protease (Roche) and phosphatase inhibitors (Roche). Cell lysates were sonicated for three cycles of 15 s pulse with 5 s interval on ice. Cell lysates were mixed gently on a rotary suspension mixer at 4°C for 2 h and centrifuged at 16,000 ***g*** for 15 min before supernatant was collected. Protein concentration was determined by performing Pierce 660 nm Protein Assay (Thermo Fisher Scientific) according to the manufacturer's instructions. Proteins (30 µg/sample) were sequentially reduced and alkylated then cleaned up using the SP3 method (Hughes et al., 2019), followed by digestion with 1.2 µg proteomic-grade trypsin/rLysC (Promega) overnight at 37°C.

### Mass spectrometry (MS) – data-independent acquisition (DIA)

Peptide samples were analyzed by nanoflow high-performance liquid chromatography–MS/MS using an Ultimate 3000 nano RSLC system (Thermo Fisher Scientific) coupled with a Q-Exactive HF mass spectrometer fitted with a nanospray Flex ion source (Thermo Fisher Scientific) and controlled using Xcalibur software (version 4.3). Approximately 1 µg of each sample was injected and separated using a 120-min segmented gradient by preconcentration onto a 20 mm×75 µm PepMap 100 C18 trapping column then separation on a 250 mm×75 µm PepMap 100 C18 analytical column at a flow rate of 300 nl/min and held at 45°C. MS Tune software (version 2.9) parameters used for data acquisition were as follows: 2.0 kV spray voltage, S-lens RF level of 60 and heated capillary set to 250°C. MS1 spectra (390-1240 m/z) were acquired at a scan resolution of 120,000 in profile mode with an automatic gain control (AGC) target of 3×10^6^ and followed by sequential MS2 scans across 26 DIA×25 amu windows over the range of 397.5-1027.5 m/z, with 1 amu overlap between sequential windows. MS2 spectra were acquired in centroid mode at a resolution of 30,000 using an AGC target of 1×10^6^, maximum injection time of 55 ms and normalized collision energy of 27.

### MS raw data processing and statistical analysis

DIA MS raw files were processed using Spectronaut software (version 14.8, Biognosys AB). A project-specific library was generated using the Pulsar search engine to search the DIA MS2 spectra against the *Homo sapiens* UniProt reference proteome concatenated with common contaminants (comprising 20,455 entries, September 2018). With the exception that single-hit proteins were excluded, default (Biognosys factory) settings were used for both spectral library generation and DIA data extraction. For library generation, these included N-terminal acetylation and methionine oxidation as variable modifications and cysteine carbamidomethylation as a fixed modification, up to two missed cleavages allowed and peptide, protein and peptide spectrum match thresholds set to 0.01. Mass tolerances were based on first-pass calibration and extensive calibration for the calibration and main searches, respectively, with correction factors set to 1 at the MS1 and MS2 levels. Targeted searching of the library based on ion current extraction deployed dynamic retention time alignment with a correction factor of 1. Quantification was based on the MaxLFQ algorithm for derivation of inter-run peptide ratios, followed by cross-run normalization based on median peptide intensity.

Differential abundance of proteins between genotypes at different time points was determined using one-way ANOVA followed by post-hoc test with unpaired two-tailed Student's *t*-test with a permutation-based false discovery rate set at 0.05 and S0=0.1 to exclude proteins with very small difference between means (Perseus software version 1.6.14.0, http://www.coxdocs.org/doku.php?id=perseus:start). PCA plot of the isogenic CLN3 neuronal proteome was generated in Spektronaut software (Biognosys, Schlieren, Switzerland). UniProt accessions for proteins that were significantly increased or decreased in abundance in CLN3 neurons compared to CLN3-Cor neurons were uploaded to DAVID Bioinformatics Resource 6.8 (https://david.ncifcrf.gov/) to retrieve the enriched KEGG pathways. KEGG pathways with *P*<0.05 after Benjamini–Hochberg correction were considered significant. The MS proteomic data for this project have been deposited to the ProteomeXchange Consortium via the PRIDE (Perez-Riverol et al., 2022) partner repository with the dataset identifier PXD032191.

### Microelectrode array

NSCs from CLN3 and CLN3-Cor were differentiated into neurons in parallel (see ‘Neural differentiation’ section). At DIV 0, 150,000 cells were replated as a 75 µl drop directly onto the PLO/laminin-coated electrode array of each well of 24-well MEA plates (MultiChannel Systems, Germany), in which each well was fitted with 12 PEDOT-coated gold electrodes. Eight wells per independent differentiated culture were used for each genotype (*n*=3 independent neuronal differentiations). After 30 min, 500 µl neuronal maturation medium was added gently to each well. Cells were incubated at 37°C, 5% CO_2_ for 4 days before the first MEA recording at DIV 4. Half-medium exchanges were performed every 2-3 days. On experimental days, the MEA plate was removed from the incubator, placed on the MEA heated stage (MultiChannel Systems, Germany) and maintained at 37°C throughout the duration of data acquisition. Plates were left to equilibrate for 2 min prior to starting recordings. Medium exchanges were performed after recording. Neuronal activity from each well was recorded with a MultiWell-MEA-system (MultiChannel Systems, Germany). MEA recordings were acquired in Multiwell-Screen Version 1.11.7.0, low-pass filtered at 3500 Hz, high-pass filtered at 1 Hz and sampled/digitized at 20 kHz. Spontaneous network activity was recorded for 5-min intervals, once per day, from DIV 4 to DIV 42. Neuronal activity was analyzed using MultiWell-Analyzer Version 1.8.7 and a customized R script. Wells containing electrodes that detected ≥10 spikes/min with minimum amplitude of 20 µV were considered active wells. Wells with at least one active electrode were included in analysis. The adaptive spike detection threshold for each electrode was set at five times the standard deviation of the baseline noise level during the first second of reading for each electrode with 1 s binning. Burst activity was detected using the following threshold parameters: maximum interval to start burst, 50 ms; maximum interval to end burst, 50 ms; minimum interval between bursts, 100 ms; minimum duration of burst, 50 ms; and minimum spike count in burst, 4 (Fig. S9A). Network bursts were defined as synchronous network spikes and bursts in which three or more electrodes (out of a total of 12) capture activity simultaneously. MEA parameters and their definitions are shown in Table S4. The values from each active electrode in each active well were averaged to determine a ‘well average’ for further analysis.

### Statistical analyses

All statistical analyses, unless otherwise indicated, were performed using R (https://www.r-project.org/), and *P*<0.05 was considered significant. We used the ‘performance’ R package (Lüdecke et al., 2021) to check regression assumptions as appropriate, which informed the modeling choices outlined below. Data from western blot, electron microscopy and RT-qPCR studies were modeled using linear mixed effects models in the lme4 package (Bates et al., 2015). To account for pseudoreplication, we fitted random intercepts (assumed to be normally distributed) for the clustering on batch. We used Tukey's method with a correction for multiple comparisons to estimate differences between cell lines.

We estimated the mean DQ-BSA puncta area normalized to cell area using mixed-effects beta regression with the glmmTMB (Brooks et al., 2017) package for R. Beta regression was appropriate because the dependent variable was a proportion and thus bounded by 0 and 1, so to estimate confidence intervals with the appropriate coverage and bounding we assumed that the residuals were beta-distributed. The model was fitted on the logit scale. Random intercepts were fitted to account for clustering within the experimental unit of batch. Inspection of residuals suggested that an excess of zeros was associated with time, so we tested a zero-inflated beta regression model using a likelihood ratio test, conditioning the zero process on time, which was significant.

Proteomic data were analyzed as described above. Data from MEA experiments were analyzed using a generalized additive model (GAM) in the mgcv package (Wood, 2017). For each MEA feature, we smoothed the response over time (DIV) by fitting a cubic spline with a shrinkage penalty. Following inspection of Q-Q plots, we determined that the residuals were not normally distributed due to skewness, so we fitted these GAMs assuming gamma-distributed residuals.

## Supplementary Material

10.1242/dmm.049651_sup1 Supplementary information
