# Supplementary material for: Lysosomal alterations and decreased electrophysiological activity in CLN3 disease patient-derived cortical neurons
Source: Dis Model Mech. 2022 Dec 13;15(12):dmm049651. doi: 10.1242/dmm.049651 (PMC10655821; doi:10.1242/dmm.049651)
Supplement: Supplementary information [file dmm-15-049651-s1.pdf]

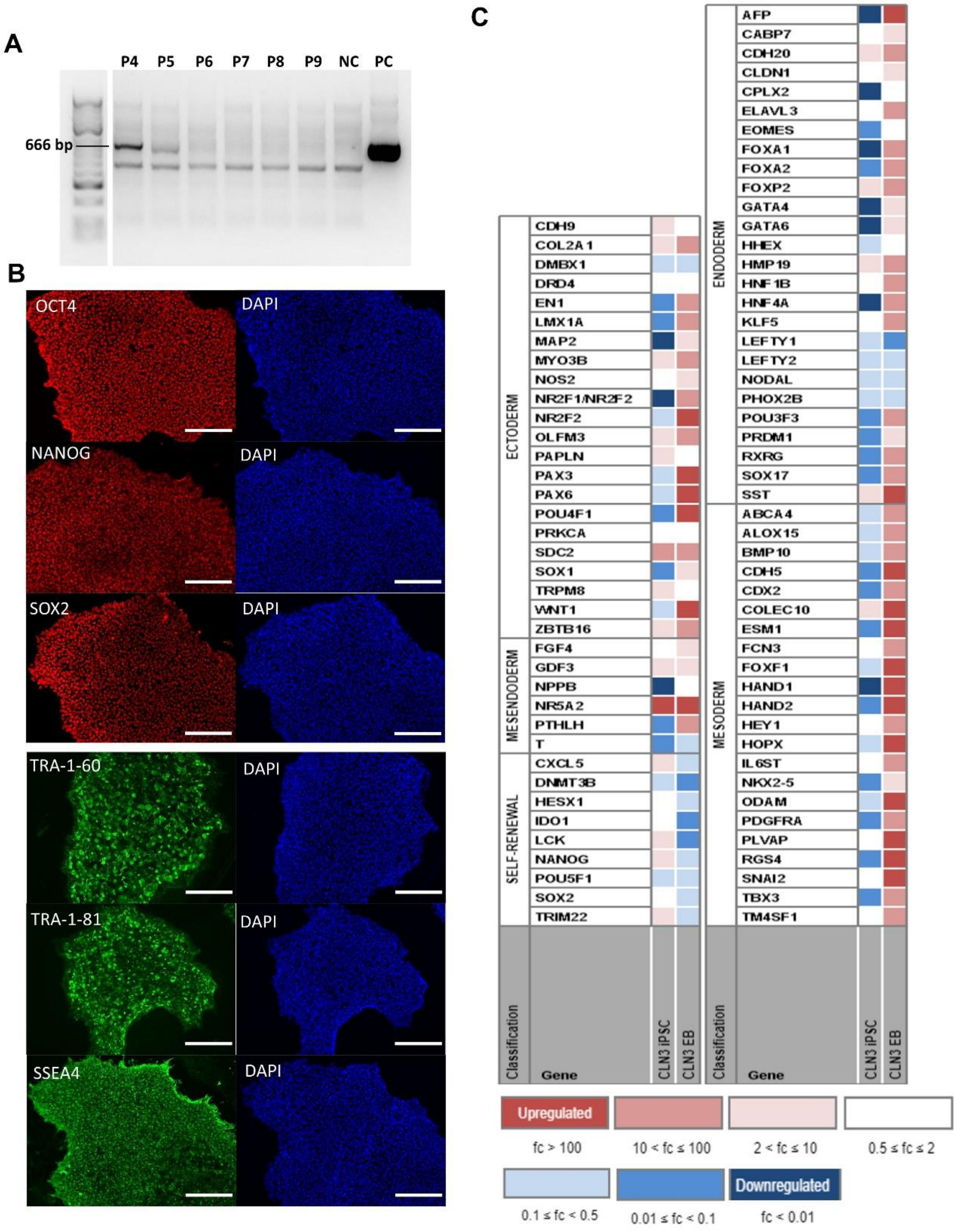

Fig. S1. Quality control assessment of patient fibroblast-derived CLN3 iPSCs. (A) Endpoint PCR showed removal of EBNA1 vector (666 bp) from CLN3 iPSCs after several passages. NC and PC represent negative control from untransfected fibroblasts and positive control from EBNA1 plasmid respectively. (B) CLN3 iPSCs displayed nuclear (OCT4,

Disease Models & Mechanisms • Supplementary information

NANOG and SOX2) and surface (TRA-1-60, TRA-1-81 and SSEA-4) markers indicating pluripotency. Scale bars: 200 µm. (C) CLN3 iPSCs showed an upregulation of pluripotent genes with downregulation of germ layer genes. Meanwhile EBs demonstrated a downregulation of pluripotent genes with upregulation of germ layer genes concurrently.

Disease Models & Mechanisms • Supplementary information

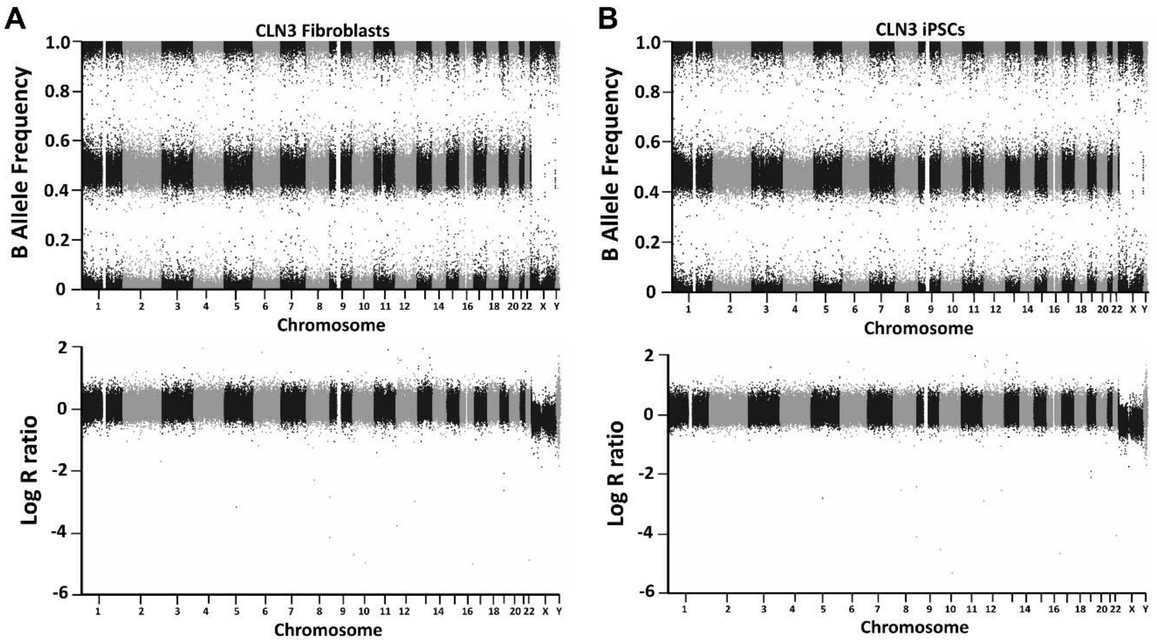

Fig. S2. Copy number variation analysis of CLN3 iPSCs. Top and bottom panels show BAF and LRR respectively for the (A) CLN3 parental fibroblasts (B) CLN3 iPSCs.

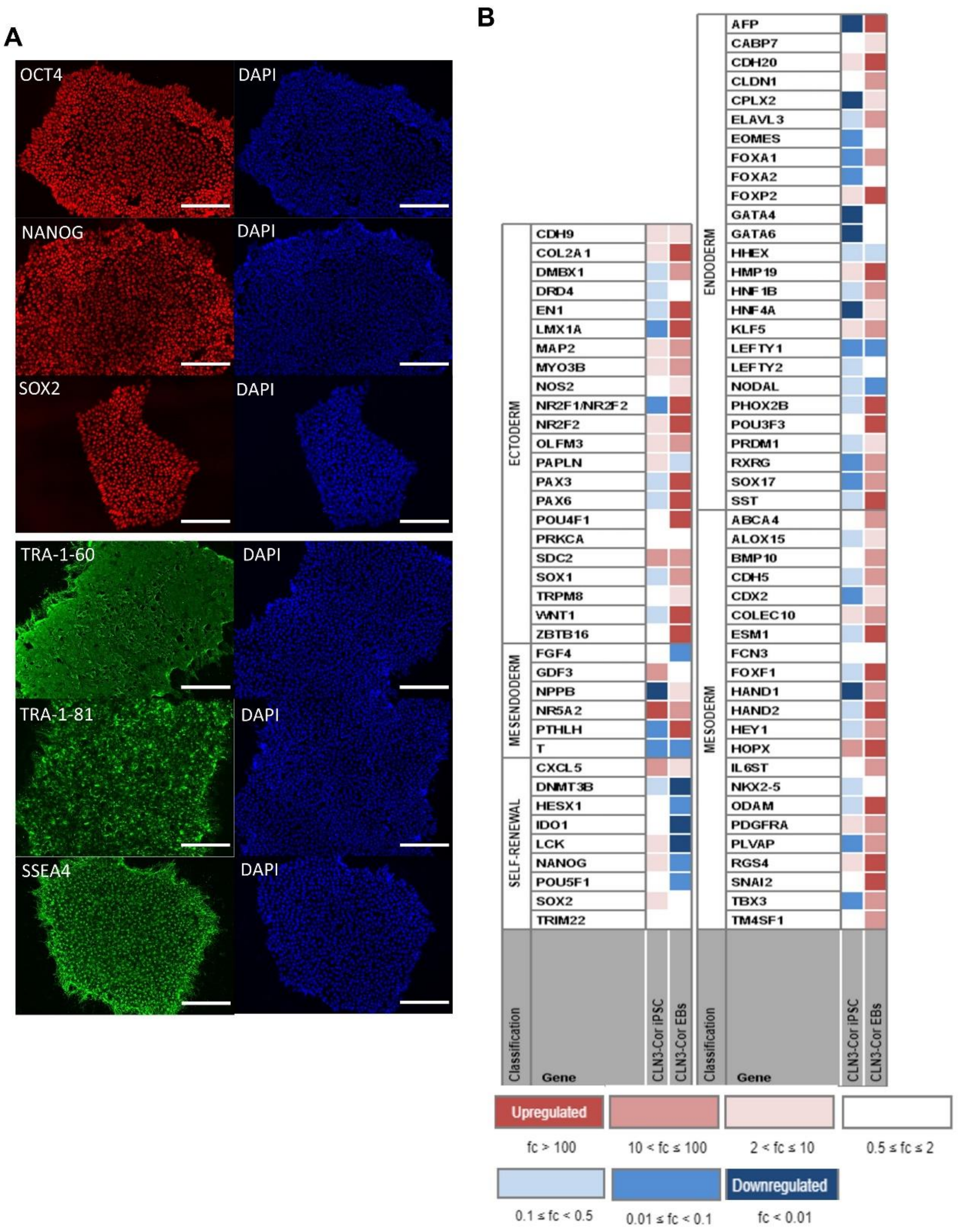

Fig. S3. Quality control assessment of CLN3-Cor iPSCs. (A) CLN3-Cor iPSCs stained positive for OCT4, NANOG, SOX2, TRA-1-60, TRA-1-81 and SSEA4. Scale bars: 200 µm. (B) Heatmap from Taqman scorecard analysis provides an overview of the expression level of pluripotent and germ layer-specific genes in CLN3-Cor iPSCs and EBs, demonstrating CLN3-Cor iPSCs were pluripotent and the EBs were able to differentiate into 3 germ layer markers.

Disease Models & Mechanisms • Supplementary information

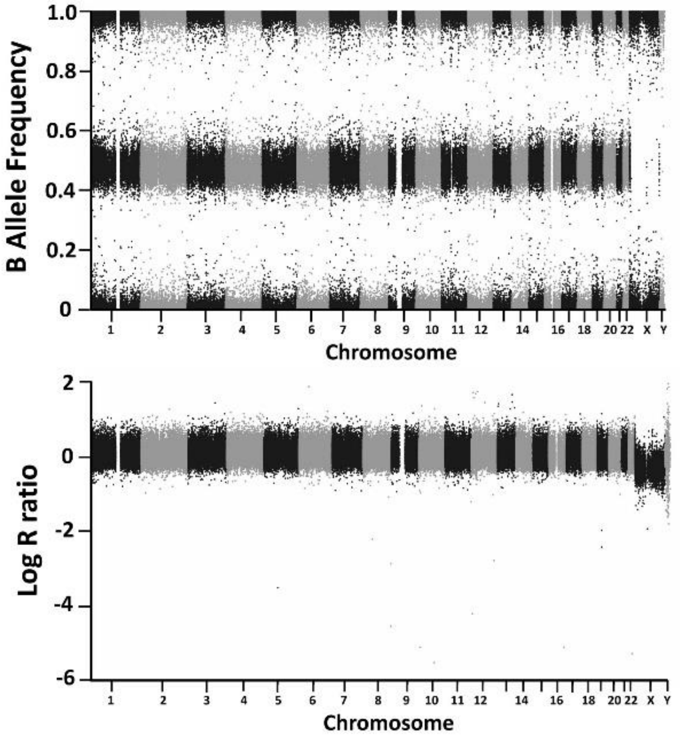

Fig. S4. Copy number variation analysis of CLN3-Cor iPSCs. Top and bottom panels show BAF and LRR respectively for the CLN3-Cor iPSCs.

Disease Models & Mechanisms • Supplementary information

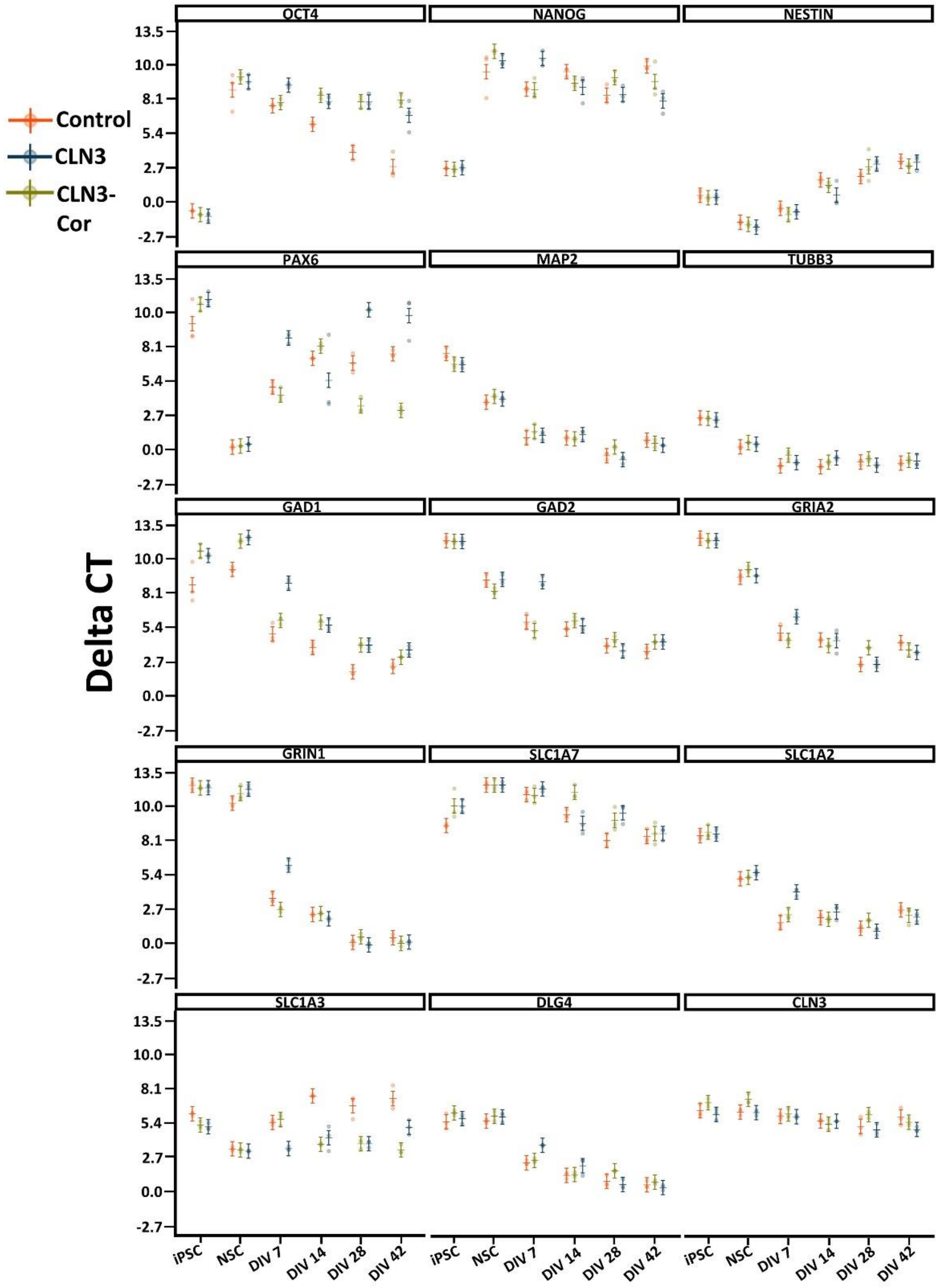

Fig. S5. Characterization of NSCs and neurons derived from isogenic CLN3 iPSCs. mRNA expression levels of genes indicating neural induction and differentiation were examined with qPCR in iPSCs, NSCs and neurons at various time points of differentiation. Data are presented as group means ± 95% confidence interval. n= 3 independent differentiated cultures per cell line per time point; linear mixed-effect model.

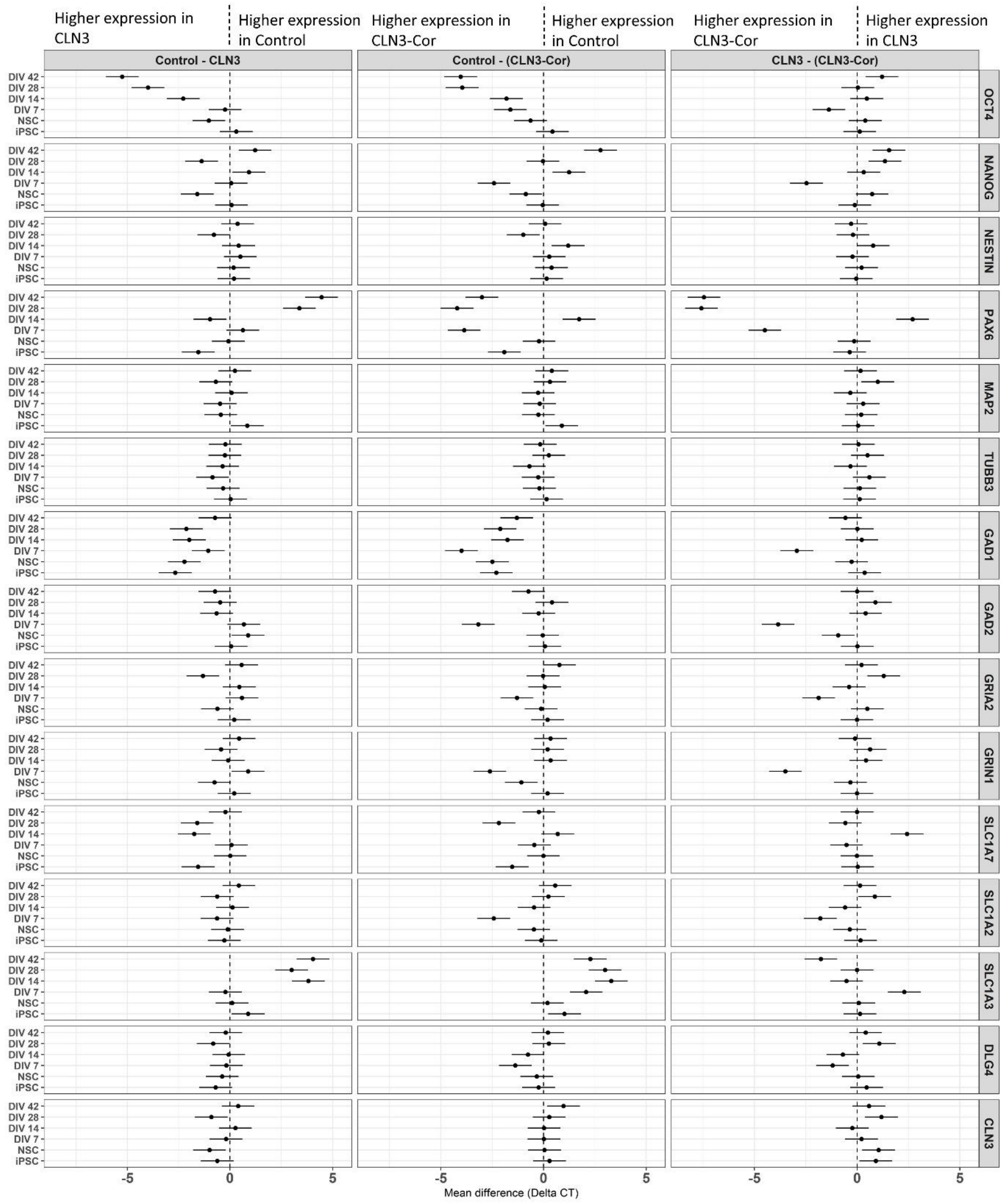

Fig. S6. Pairwise comparison of differences in mRNA expression. Forest plot depicts mean differences in mRNA expression for various genes between control, CLN3 and CLN3-Cor neurons throughout differentiation period. Data are presented as mean difference ± 95% confidence interval, *$p < 0.05$. Dotted vertical line represents line of null effect.

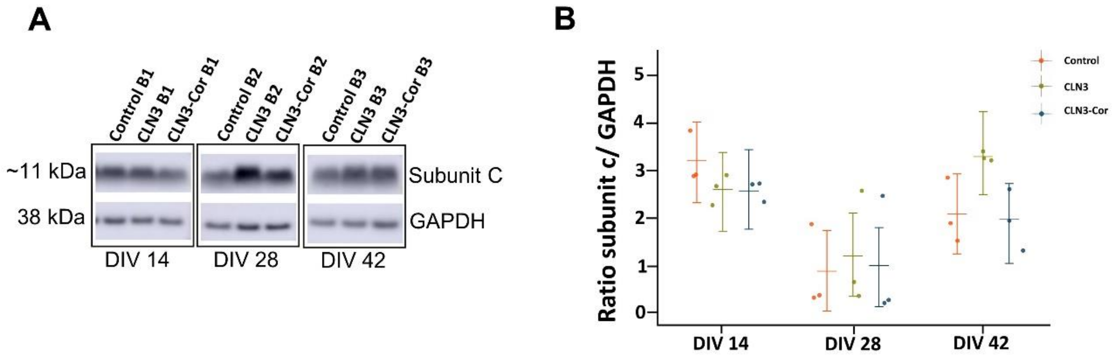

Fig. S7. Expression of subunit C in control and isogenic CLN3 neuronal cultures. (A) Western blots showing subunit C protein expression across different time points. (B) Quantitation of subunit C in both cell lines across different time points. Data are presented as group means ± 95% confidence interval. n= 3 independent differentiated cultures per cell line per time point; linear mixed effect model.

Disease Models & Mechanisms • Supplementary information

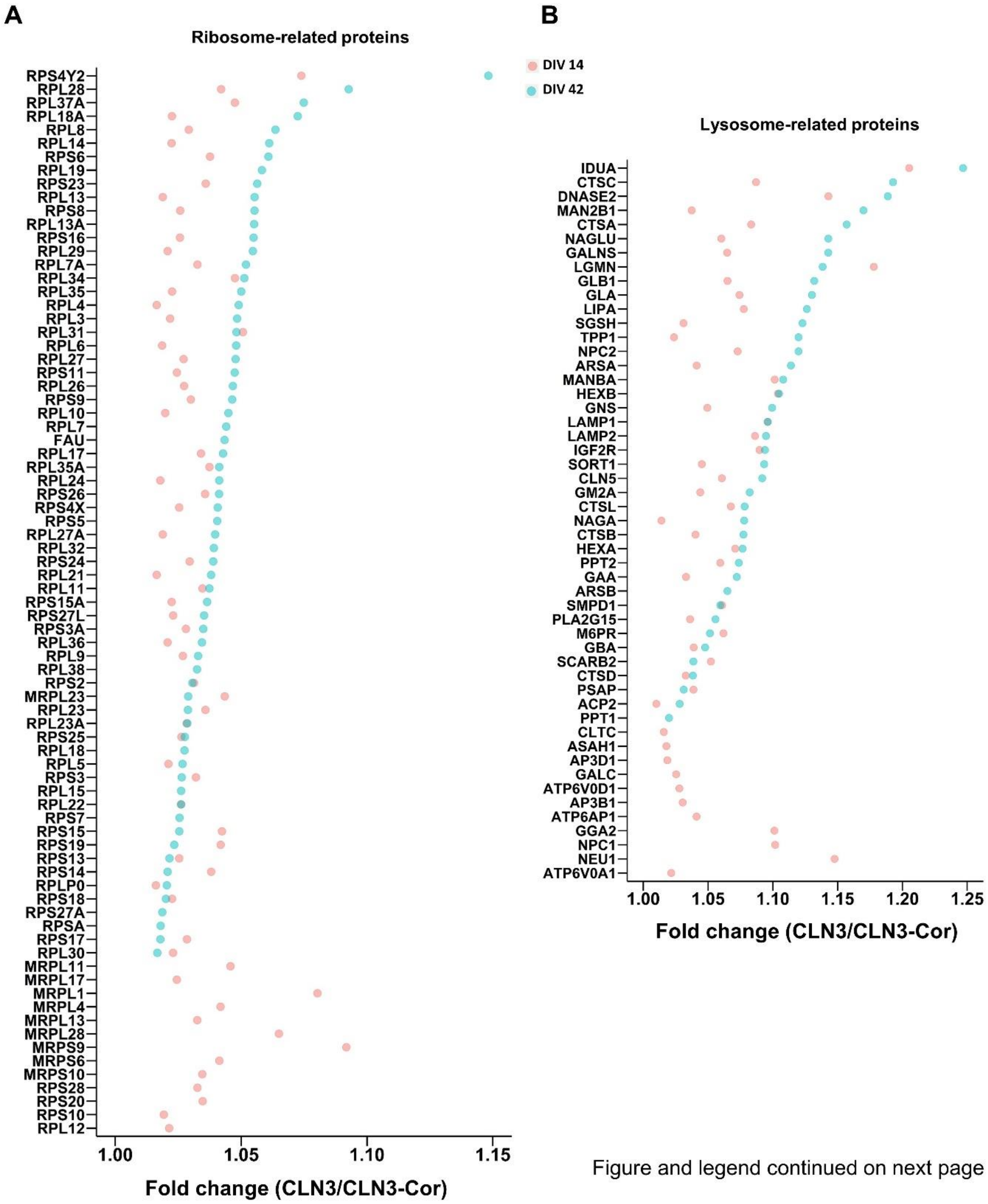

Figure and legend continued on next page

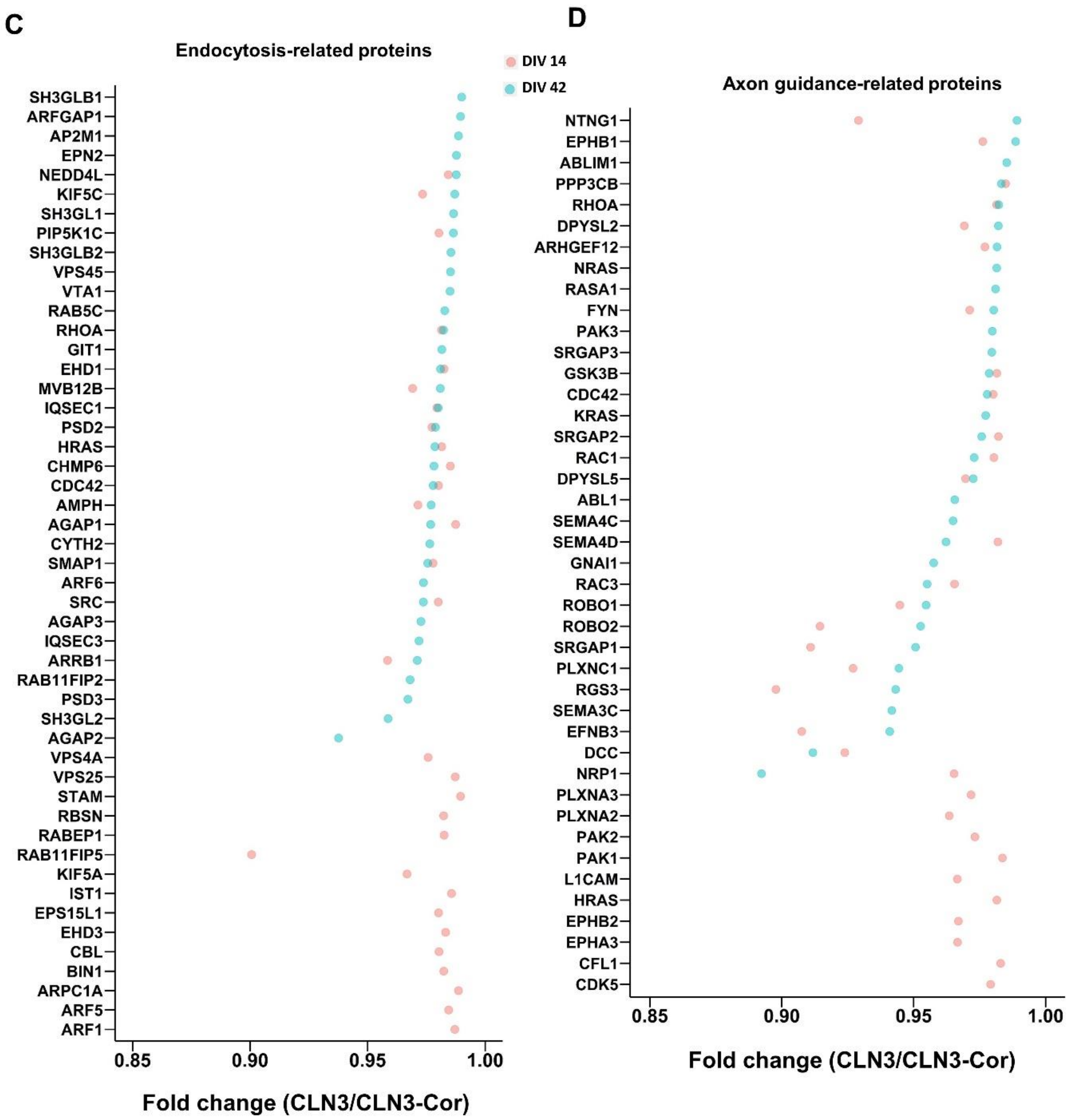

Fig. S8. Altered KEGG pathways in CLN3 neurons. Overview of mean fold change of protein expression in CLN3 neurons compared to CLN3-Cor neurons for enriched KEGG pathways related to (A) ribosome-related proteins and (B) lysosomal proteins increased in abundance (mean fold change >1), (C) endocytosis and (D) axon guidance-related proteins reduced in abundance (mean fold change <1) at DIV 14 and 42 (n=3 independent differentiated cultures per cell line per time point).

Disease Models & Mechanisms • Supplementary information

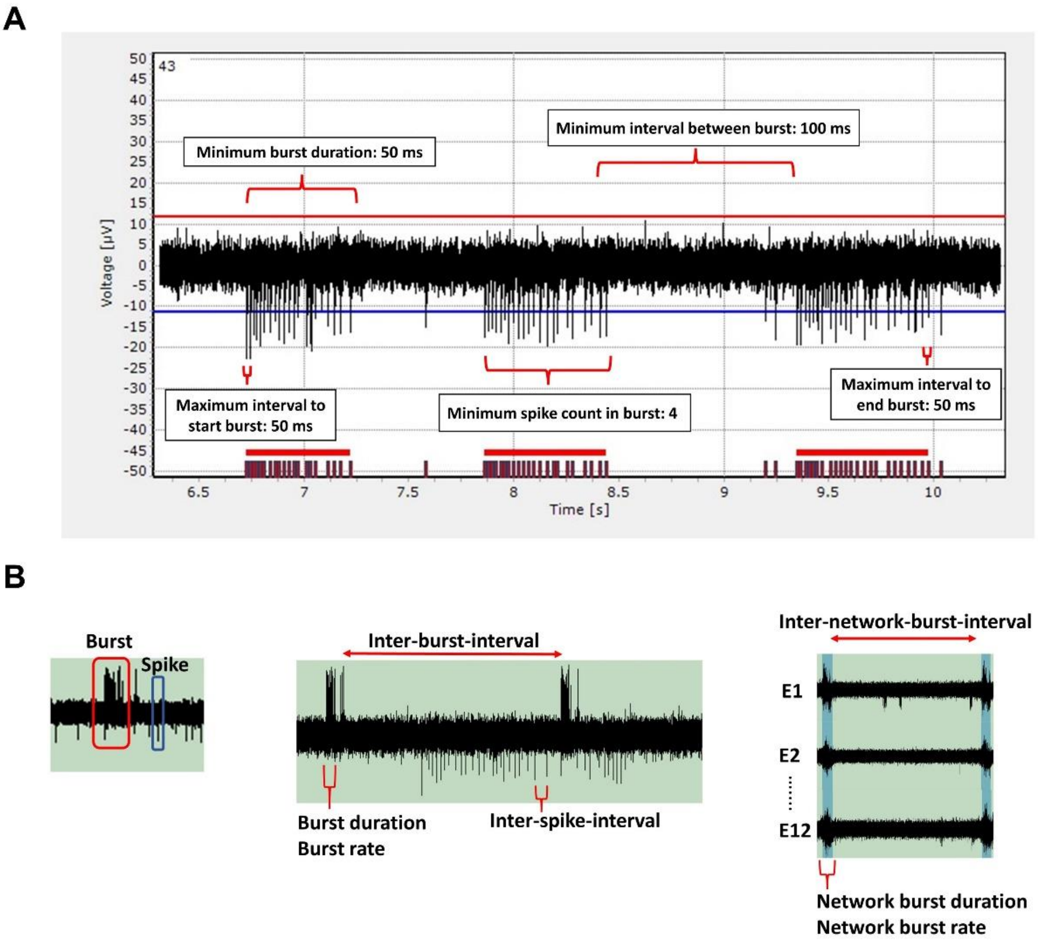

Fig. S9. MEA parameters used to measure electrophysiological activity. (A) Five threshold parameters were used to identify bursts as shown on raw data. (B) Schematic overview of extracted parameters from MEA raw data including spikes, bursts and network bursts.

Disease Models & Mechanisms • Supplementary information

Fig. S10. **Raw images for western blotting.**

1) LAMP1

DIV 14

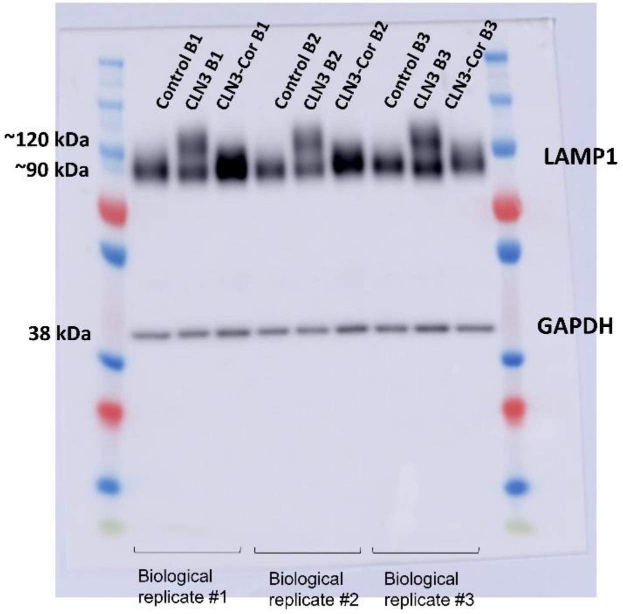

DIV 28

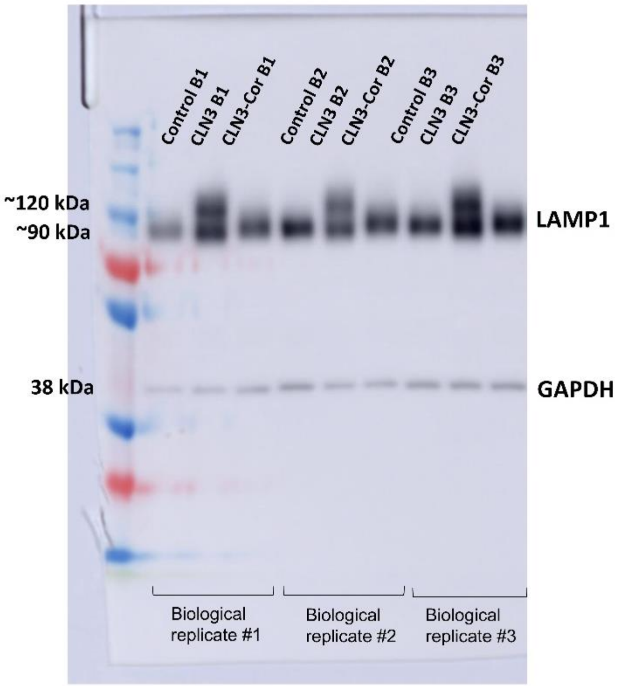

Disease Models & Mechanisms • Supplementary information

DIV 42

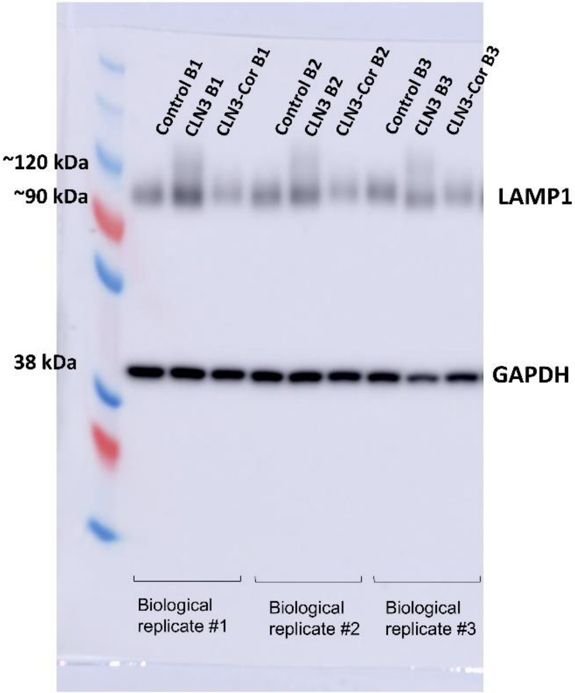

Disease Models & Mechanisms • Supplementary information

2) Subunit C

DIV 14

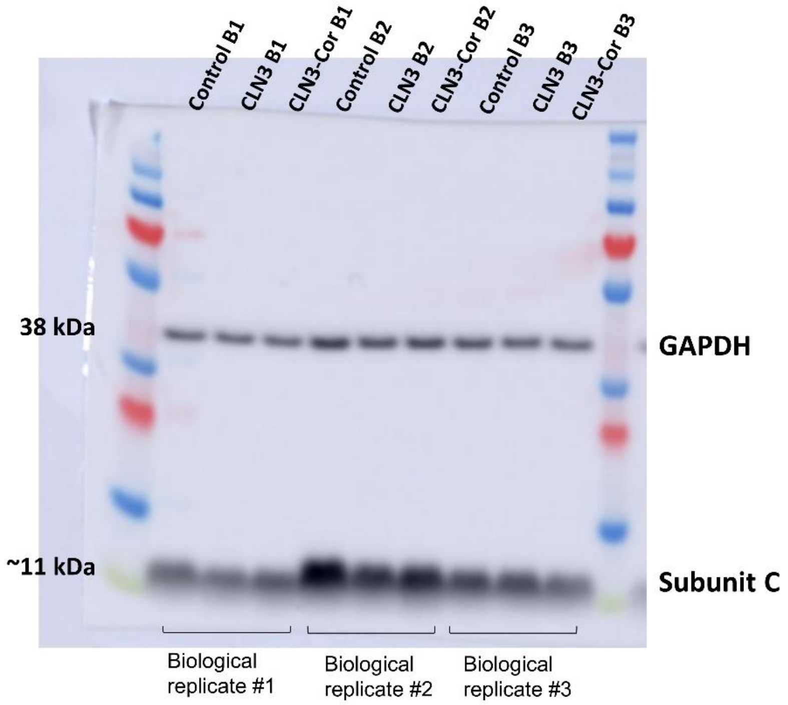

DIV 28

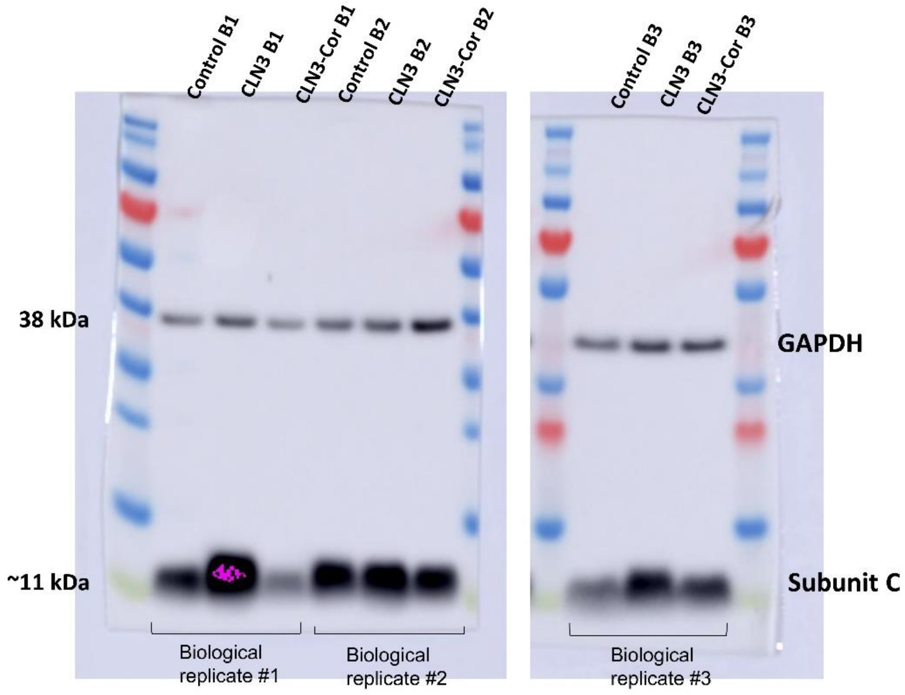

Disease Models & Mechanisms • Supplementary information

DIV 42

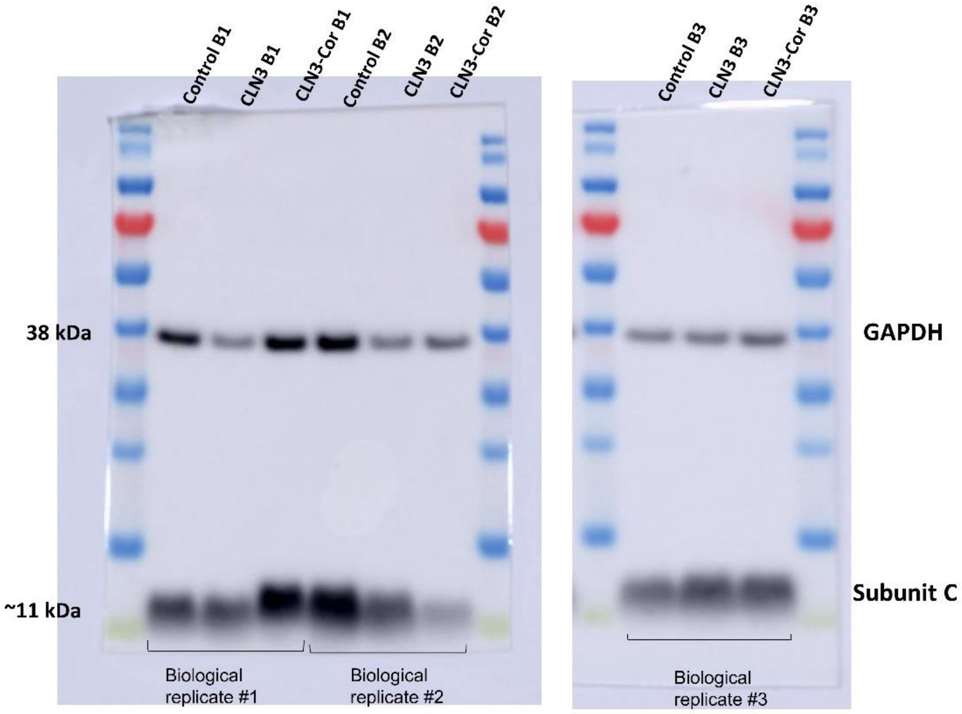

Disease Models & Mechanisms • Supplementary information

3) LAMP1 deglycosylation

Biological replicate #1

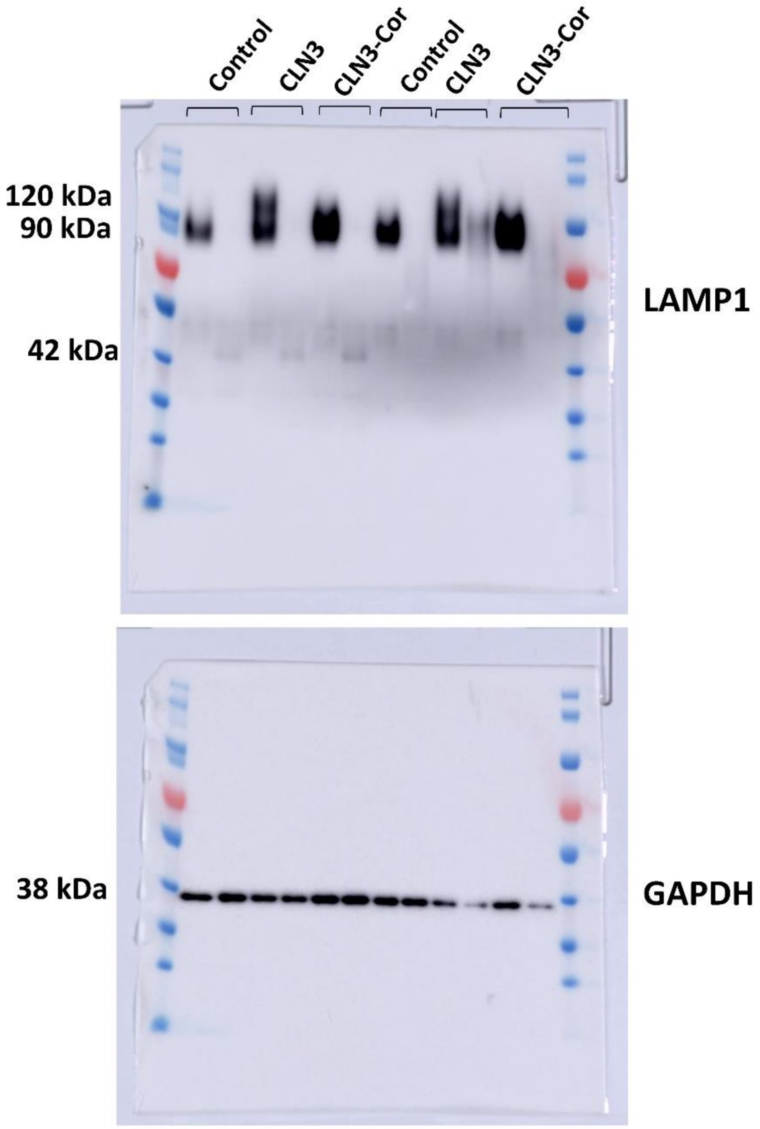

Disease Models & Mechanisms • Supplementary information

Biological replicate #2

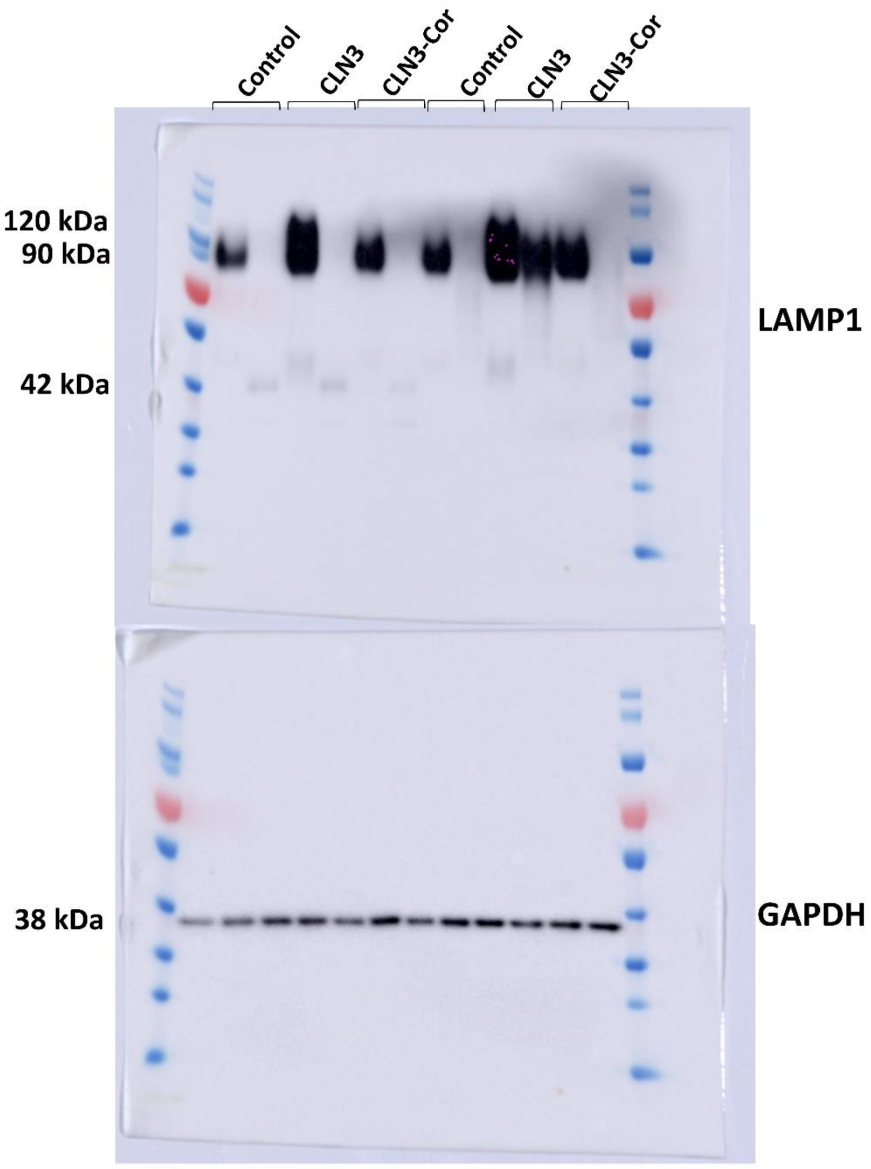

Disease Models & Mechanisms • Supplementary information

Table S1. Off-target analysis

Sequence	PAM	Gene	Chromosome	Mismatches	Modification
CAGAGTATGGACTTGAAGGA	AAG		Chr20	3	No
CCCGTTAGGGACTTGAAGGA	AAG		Chr19	3	No
CAAGGCAGGGAATTGAAGGA	AGG		Chr5	2	No
GCAGGTAGAGACTTGAAGGA	GAG		Chr2	3	No
CAATCTTGGGACTTGAAGGA	AAG		Chr12	3	No
CTAGGCAAGGACTTGAAGGA	GGG		Chr15	3	No
ACAGGTAGGGACTTGAGGGA	AAG	ENSG00000157429	Chr16	3	No
CAATGCAGGAACTTGAAGGA	GAG		Chr3	3	No
CAAGGTAGGAACTTGAAAGA	TGG		Chr2	2	No
CAAAGTAGGTATTTGAAGGA	AAG		Chr10	3	No
CCAGGTCGGGTCTTGAAGGA	TGG	ENSG00000279069	Chr17	3	No
CAAGTTTTTGACTTGAAGGA	AAG	ENSG00000182836	Chr5	4	No

Disease Models & Mechanisms • Supplementary information

Table S2. List of all oligonucleotides used in this study

Oligo name	Sequence	Region to amplify	Expected PCR band size
EBNA-1 F	ATCGTCAAAGCTGCACACAG	Sequence in EBNA-1 episomal vector	666 bp
EBNA-1 R	CCCAGGAGTCCCAGTAGTCA		
CLN3 F1	GGATGAATTAGATGGAGATTGAGG	CLN3 DNA: region spanning exons 7,8	1 kb deletion: 563 bp<br>Wild type: 1529 bp<br>HDR insertion with puromycin cassette: 2581 bp
CLN3 R1	CTCATCCTACTTCTAATCACCTTG		Wild type: 1529 bp<br>HDR insertion with puromycin cassette: 2581 bp<br>HDR insertion without puromycin cassette: 1563 bp
CLN3_SS_cdna_F1	TCTGTCTCTACGGCTGCTGTGC	CLN3 cDNA: region spanning exons 7,8	Deleted: 578bp
CLN3_SS_cdna_R1	GAACACCAGGTTGAGGCACTGC		Undeleted: 795bp

Disease Models & Mechanisms • Supplementary information

Table S3. List of Taqman assay probes for neural induction and differentiation

Marker	Gene name	Taqman Assay ID
Pluripotency	*OCT4*	Hs01895061_u1
Pluripotency	*NANOG*	Hs00415716_m1
Neuroepithelial stem cell	*NESTIN*	Hs04187831_g1
Neuroepithelial stem cell	*PAX6*	Hs01088114_m1
Mature neuron	*MAP2*	Hs00258900_m1
Microtubule	*TUBB3*	Hs00801390_s1
γ-aminobutyric acid biosynthesis enzyme	*GAD1*	Hs01065893-m1
γ-aminobutyric acid biosynthesis enzyme	*GAD2*	Hs00609534_m1
AMPA receptor	*GRIA2*	Hs00181331_m1
NMDA receptor	*GRIN1*	Hs00609557_m1
Excitatory amino acid transporter	*SLC1A7*	Hs00220404_m1
Excitatory amino acid transporter	*SLC1A2*	Hs01102423_m1
Excitatory amino acid transporter	*SLC1A3*	Hs00188193_m1
Postsynaptic density protein	*DLG4*	Hs01555370_g1
Reference gene	*EEF2*	Hs00157330_m1

Table S4. MEA parameters and description

Parameters	Description
Spike rate (Hz)	Number of spikes/second
Interspike interval (s)	Time interval between two consecutive spikes
Percentage of active electrode (%)	Percentage of number of active electrodes divided by total number of electrodes
Burst rate (bursts/min)	Number of bursts per minute
Burst duration (s)	Duration of burst
Interburst interval (s)	Time interval between two consecutive bursts
Percentage of spikes in burst (%)	Percentage of number of spikes which were transformed into bursts divided by total number of spikes
Network burst rate (bursts/min)	Number of network bursts per minute
Network burst duration (s)	Duration of network burst

Disease Models & Mechanisms • Supplementary information
